# Effectiveness of multi-component exercise in individuals with type 2 diabetes: a systematic review and meta-analysis

**DOI:** 10.7717/peerj.20146

**Published:** 2025-11-20

**Authors:** Zhiyuan Sun, Haiqing Zeng, Hualei Liu, Mengqi Hu, Xuewen Tian, Dewei Mao, Rui Zhang

**Affiliations:** 1 Research Institute of Sports Science, Shandong Sport University, Jinan, China; 2 College of Sport Science, Qufu Normal University, Qufu, China; 3 Division of Physical Education, Chinese University of Hong Kong, Shenzhen, China

**Keywords:** Multi-component, Type 2 diabetes

## Abstract

**Objective:**

This meta-analysis aimed to explore the effects of multi-component exercise interventions on glycemic and lipid metabolism, physical fitness, and cognitive function in individuals with type 2 diabetes mellitus (T2DM).

**Methods:**

From inception to December 28, 2024, PubMed, Web of Science, Cochrane, and Elsevier databases were systematically searched for randomized controlled trials (RCTs) investigating multi-component exercise interventions for T2DM. A total of 37 articles, comprising 3,201 participants, were included. Primary and secondary outcome measures were categorized, summarized, and analyzed using RevMan 5.4 software.

**Results:**

Compared to control groups, multi-component exercise interventions produced statistically significant improvements across all measured outcomes in individuals with T2DM: (1) Glycemic control: HbA1c (standard mean difference (SMD) = −0.52, 95% confidence interval (CI) [−0.76 to −0.28]); fasting blood glucose (SMD = −0.53, 95% CI [−0.93 to −0.12]). (2) Lipid metabolism: high density lipoprotein (HDL) (SMD = 0.32, 95% CI [0.21–0.44]); low density lipoprotein (LDL) (SMD = −0.21, 95% CI [−0.33 to −0.09]); triglycerides (SMD = −0.18, 95% CI [−0.30 to −0.06]). (3) Physical fitness: upper limb strength (SMD = 0.67, 95% CI [0.51–0.83]); lower limb strength (SMD = 0.56, 95% CI [0.10–1.02]); peak oxygen consumption (SMD = 0.62, 95% CI [0.31–0.93]); body mass index (BMI) (SMD = −0.38, 95% CI [−0.67 to −0.09]). (4) Cognitive function: overall cognitive performance (SMD = 0.34, 95% CI [0.18–0.50]). (5) Quality of life: vitality (SMD = 0.37, 95% CI [0.09–0.64]); physical functioning (SMD = 0.48, 95% CI [0.20–0.75]); mental health (SMD = 0.35, 95% CI [0.07–0.63]); general health (SMD = 0.34, 95% CI [0.06–0.61]). Quality assessment indicated that the included studies were of high overall quality. Egger’s regression analysis did not reveal significant publication bias.

**Conclusions:**

Multi-component exercise interventions significantly improved glycemic and lipid metabolism, physical fitness, and cognitive function in individuals with T2DM. These findings support the clinical value of incorporating multi-component exercise programs—particularly those performed at least three times per week and lasting 6 months or longer—into diabetes management strategies.

## Introduction

Diabetes is the most prevalent metabolic disease globally, with projections indicating that the number of individuals affected will reach to 1.3 billion by 2050 (GBD 2021 Diabetes Collaborators, 2023). Type 2 diabetes mellitus (T2DM) accounts for 90–95% of all diabetes cases (Pedroso et al., 2021). The prevalence of diabetes and its associated complications poses a significant threat to global health (Zheng, Ley & Hu, 2018) compromising the self-management capabilities of individuals with T2DM and placing additional pressure on national healthcare systems. While pharmacological treatments can manage hyperglycemia, their long-term effectiveness is limited (Raveendran, Chacko & Pappachan, 2018), and improper usage may double the risk of dementia among T2DM patients (Mcmillan et al., 2018). Given this context a multidisciplinary approach is urgently needed to protect the health of the global T2DM population.

Clinical practice has indicated that exercise, as a non-pharmacological intervention, is effective in ameliorating glycolipid metabolic dysregulation in patients with T2DM (Lin et al., 2023). Common exercise modalities include aerobic, resistance, and multi-component exercises. Multi-component exercise involves interventions using two or more types of exercise (Suzuki et al., 2012), such as a combination of aerobic and resistance training, balance/coordination, and flexibility exercises. Studies show that multi-component exercise outperformed single-modality exercise in improving glycemic control (Silveira-Rodrigues et al., 2021), lipid levels (Schwingshackl et al., 2014), and inflammatory status (Hopps, Canino & Caimi, 2011) among patients with T2DM. Beyond metabolic effects, multi-component exercise can induce beneficial neural adaptations through neuroplasticity and various signaling pathways, leading to enhanced cognitive functions, attention, and verbal fluency. Additionally, it may exert restorative effects on mild cognitive impairment (MCI) in the elderly (Umpierre et al., 2013). Specifically, single-mode exercises have unique mechanisms that improve the health status of patients with T2DM (Zhang et al., 2024). Compared to single-mode exercises (such as aerobic or resistance training), the benefits of multi-component exercise seem to stem not only from an increase in total exercise volume but also from the synergistic effects generated by combining different types of exercise, which provides greater advantages (Sigal et al., 2007; Balducci et al., 2007). For example, resistance training increases muscle mass and glycogen storage (Knuiman, Hopman & Mensink, 2015), while aerobic exercise enhances mitochondrial function and glucose utilization (Wang et al., 2020). Together, these effects help improve blood glucose control by reducing insulin resistance and enhancing glucose metabolism (Yavari et al., 2012). In terms of cardiovascular health, aerobic exercise improves cardiac output, vascular function, and endothelial health (Al-Mhanna et al., 2023), while resistance training contributes to better arterial stiffness and blood pressure regulation (Okamoto, Masuhara & Ikuta, 2013). By combining these benefits, multi-component exercise can improve cardiovascular health and enhance cardiac function. Global recommendations for multi-component exercise in individuals with T2DM have been substantial (Care, 2018). Although current systematic reviews have confirmed that multi-component exercise is superior to single-mode exercise in improving blood glucose (Sun et al., 2024a), inflammation (Xing et al., 2022), and cognitive function (Sun et al., 2024b), patients with T2DM face not only dysregulated glucose and lipid metabolism but also complications such as sarcopenia (Anagnostis et al., 2020) and impaired cardiopulmonary function (Pugliese et al., 2023). However, there is still a lack of systematic reviews evaluating the overall effects of multi-component exercise on the health status of elderly patients with T2DM, indicating that further research is needed to comprehensively assess its overall benefits. Compared to previous meta-analyses, this review provides a detailed compilation and summary of the effects of multi-component exercise interventions on improving HbA1c, blood glucose, lipid metabolism, cognitive function, and physical fitness in individuals with T2DM, offering theoretical support for the use of multi-component exercise in preventing and improving the overall function of patients with T2DM.

## Methods

This study adhered to the Cochrane Handbook for Systematic Reviews of Interventions and the Preferred Reporting Items for Systematic Reviews and Meta-Analyses (PRISMA) guidelines (Moher et al., 2009), This study was registered on the International Prospective Register of Systematic Reviews (PROSPERO), and the registration number is CRD42024527965.

### Literature search strategy

From inception to December 28, 2024, PubMed, Web of Science, Cochrane, and Elsevier databases were systematically searched for randomized controlled trials (RCTs) investigating multi-component exercise interventions for diabetes. Independent searches were conducted by two researchers in PubMed, Web of Science, Cochrane, and Elsevier databases, using a Boolean combination of terms related to study type (randomized controlled trials), target population (T2DM), and intervention (multi-component exercise). Additional manual searches were conducted to ensure completeness by reviewing references of included studies. The search strategy details are shown in Table 1.

**Table 1:** The search terms of the literature included in this study.

Group	Subject word
1	Randomized controlled trial, controlled clinical trial, randomized, randomised, trial, groups, RCT
2	Associated movement, combined training, multi-mode motion, multi-mode locomotion, multi-modal motion, various forms of exercise, multi-component exercise, aerobic combined with resistance exercise
3	Diabetes, type 2 diabetes mellitus, type 2 diabetes, type 2 diabetic mellitus, diabetes mellitus, 2 diabetes mellitus, type 2 diabete, type 2 diabetic, T2D

### Inclusion and exclusion criteria

Following the Population, Intervention, Comparison, Outcome, and Study (PICOS) framework, this meta-analysis considered study design, population, intervention measures, and outcomes, with language restrictions. Inclusion criteria: (1) Subjects: patients with T2DM, diagnosed according to standardized guidelines by the American Diabetes Association (Standards of Medical Care in Diabetes, SOC), World Health Organization (WHO), and International Diabetes Federation (IDF); (2) Intervention: combinations of two or more types of exercise; (3) Outcomes: HbA1c, glycemia, lipid metabolism, physical fitness, cognitive function, quality of life; (4) Study design: RCTs published in English, with no significant pre-intervention differences between experimental and control groups; (5) Control group: Participants receiving no exercise intervention, usual care only, or minimal physical activity (*e.g*., stretching, relaxation techniques, or health education sessions) that is not expected to significantly affect metabolic or physical outcomes. Exclusion criteria: (1) Studies irrelevant to the research theme (non-T2DM or single-mode exercise interventions); (2) Non-RCTs; (3) Duplicates or overlapping studies; (4) Studies with incomplete outcome data, making it impossible to convert mean differences for meta-analysis.

### Literature screening and data extraction

Two researchers (ZHQ and SZY) independently screened titles and abstracts using EndNote X9, followed by full-text assessments to determine inclusion. Discrepancies in data extraction were resolved through consensus discussions between the two researchers (ZHQ and SZY). If consensus could not be reached, a third researcher (LHL) made the final decision. Data extraction information includes the following four aspects. (1) basic information: first author, year of publication; (2) study characteristics: sample size and age of experimental and control groups, intervention measures, period, frequency; (3) research outcomes: primary outcomes were changes in HbA1c, glycemia, and lipid metabolism; secondary outcomes were changes in physical fitness, cognitive function; (4) for continuous outcomes, mean differences between baseline and endpoint for intervention and control groups were converted into effect sizes. The final data extraction table was completed with the intervention of a third researcher (LHL).

### Risk of bias and quality assessment

A total of 37 studies were included and assessed for bias and quality using the Cochrane Collaboration’s tool for assessing the risk of bias in randomized trials. Each study underwent assessment based on various criteria, including sequence generation, allocation concealment, blinding of participants and personnel, blinding of outcome assessment, completeness of outcome data, selective reporting, and other biases. Subsequently, studies were categorized according to their risk of bias (low, high, uncertain) and were independently evaluated for the quality of evidence pertaining to each outcome. Discrepancies were discussed with a third researcher until consensus was reached. Based on the assessment, included studies were graded as: (A) satisfying five or more low-risk items; (B) three to four low-risk items; (C) two or fewer low-risk items.

### Statistical methods

The outcome data of the included studies were analyzed using Revman 5.4. All outcomes were continuous variables and standardized mean differences (SMD) were used to combine effect sizes. Heterogeneity among studies was assessed using a chi-squared test, with *P* < 0.10 indicating significant heterogeneity. Additionally, I^2^ was used to assess the degree of heterogeneity; If I^2^ > 50%, significant heterogeneity is considered present, and a random-effects model is employed to pool effect sizes. The robustness of the results was then assessed by excluding low-quality studies or those that contributed substantially to the heterogeneity. If heterogeneity was low or unclear, we applied a fixed-effects model. Furthermore, for outcome measures with more than 10 studies, a fixed-effects model was employed for meta-analysis. Publication bias for each outcome was evaluated both qualitatively and quantitatively using Egger’s test and funnel plots. Effect sizes for all outcomes were pooled and assessed with 95% confidence intervals (95% CI), with statistical significance level set at 0.05.

## Results

### Literature search results

**Figure 1:**
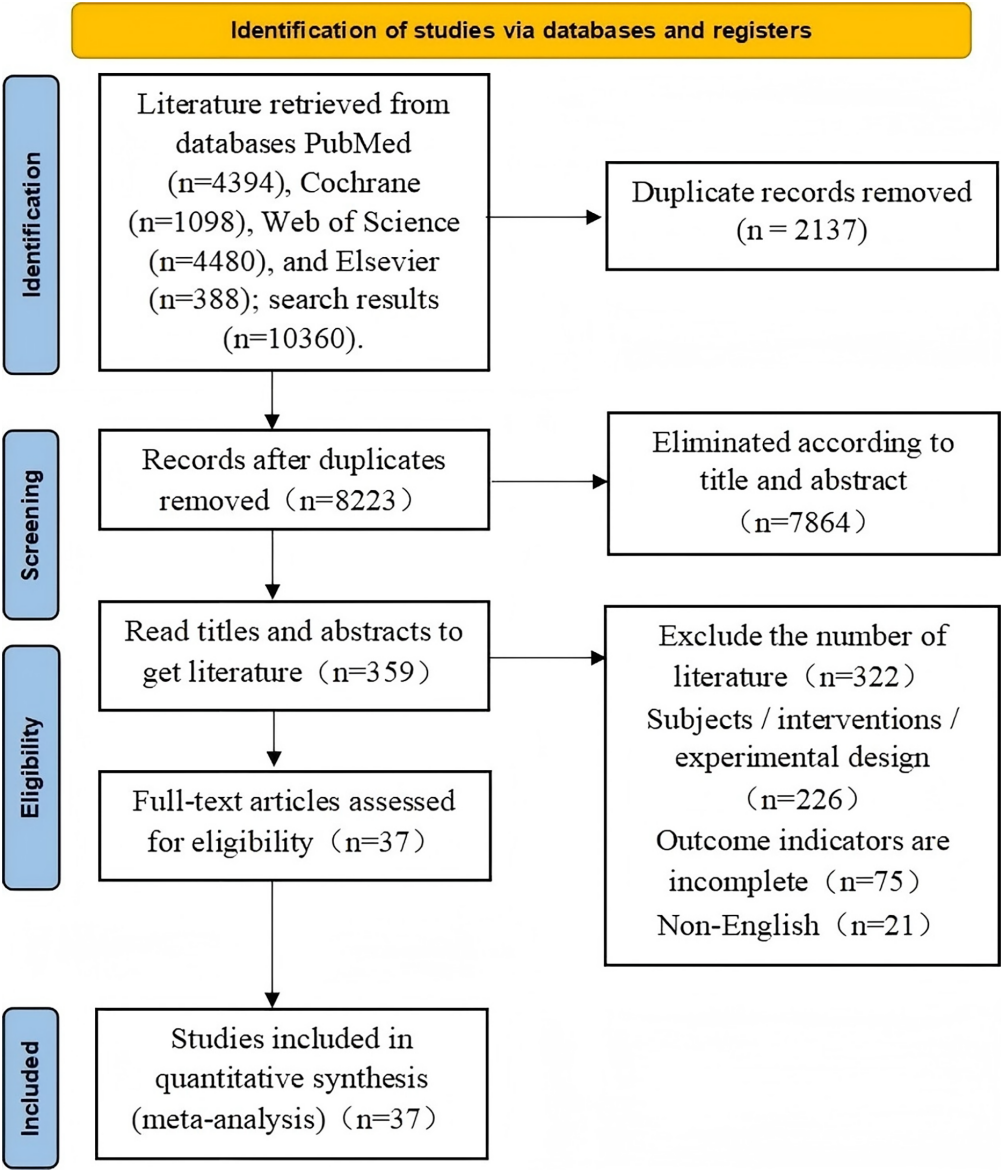
Flow diagram of study selection procedure.

The initial search yielded 10,360 articles, which were imported into EndNote X9 for deduplication, leaving 8,223 articles. Titles and abstracts were further screened, excluding 7,864 unrelated articles, leaving 359 articles. Full-text screening identified 37 articles that met the criteria for meta-analysis. The specific screening stages and criteria are illustrated in Fig. 1.

### Characteristics of included studies

The 37 articles included in this review utilized multi-component exercise interventions, encompassing a total of 3,201 participants. Sex proportion was reported across all studies,with 1,535 males and 1,666 females included. Moreover, 34 articles provided the average ages for both control and intervention groups, with ages ranging from 50 to 89 years. The exercise interventions varied, with 25 articles involving combined aerobic and resistance training, three articles incorporating aerobic resistance training with balance exercises, two featuring water-based aerobic and resistance training, two including mind-body exercises, and one each of the following: aerobic resistance training combined with leisure activities, folk dance combined with resistance training, sensory motor exercises combined with gait training, walking combined with resistance training, and strength training combined with balance exercises. In terms of exercise frequency, 30 articles required an exercise frequency of ≥3 times per week, while six articles set a minimum of twice per week. The duration of the interventions was less than 6 months in 20 articles and 6 months or longer in 17 articles. Detailed characteristics of the included studies are summarized in Table 2.

**Table 2:** Characteristics of the included studies.

Author (year)	Subjects		Intervention measures				Outcome
	Male/female	Age (years)	Intervention exercise	Control group	Frequency (times/week)	Duration (week/month)	
Sigal et al. (2007)	81/46	C: 54.8	Aerobic exercise	Sedentary	3	6 months	HbA1c, Lipid metabolis, BMI
		I: 53.5	Resistance exercise				
Church et al. (2010)	40/77	C: 58.6	Aerobic exercise	Stretching and relaxation	3	9 months	HbA1c, Peak oxygen uptake
		I: 55.4	Resistance exercise				
Lambers et al. (2008)	13/15	C: 57.6	Aerobic exercise	No intervention	3	3 months	HbA1c, Lipid metabolis, Physical fitness, Quality of life
		I: 55.8	Resistance exercise				
Tomas-Carus et al. (2016)	17/13	C: 58.9	Aerobic exercise	Sedentary	3	12 weeks	HbA1c, Quality of life
		I: 59.9	Resistance exercise				
Seyedizadeh, Cheragh-Birjandi & Hamedi Nia (2020)	0/22	C: 60.81	Aerobic exercise	No intervention	3	8 weeks	Muscle strength
		I: 56.69	Resistance exercise				
Johansen et al. (2017)	51/47	C: 56.6	Aerobic exercise	Standard care	5–6	12 months	HbA1c, Lipid metabolis, Physical fitness
		I: 53.6	Resistance exercise				
Macdonald et al. (2021)	51/47	C: 56.6	Aerobic exercise	Standard care	5–6	12 months	Quality of life
		I: 53.6	Resistance exercise				
Aylin et al. (2009)	27/9	C: 56.06	Aerobic exercise	No intervention	≥2	8 weeks	HbA1c, Blood sugar, Lipid metabolis, BMI,
		I: 51.39	Resistance exercise				
Myers et al. (2013)	30/47	C: 58.1	Aerobic exercise	Sedentary	3	9 months	Quality of life
		I: 56.9	Resistance exercise				
Balducci et al. (2010)	329/234	C: 58.8	Aerobic exercise	Individual consultation	2	12 months	HbA1c, Blood sugar, Lipid metabolis, Physical fitness
		I: 58.8	Resistance exercise				
Reid et al. (2010)	71/38	C: 55.2	Aerobic exercise	Sedentary	3	6 months	Quality of life
		I: 53.3	Resistance exercise				
Stomby et al. (2017)	71/7	C: 59.0	Aerobic exercise	Dietary therapy	3	12 weeks	HbA1c, Blood sugar, Lipid metabolis, Physical fitness, Overall cognitive function
		I: 61.0	Resistance exercise				
Callisaya et al. (2017)	26/24	C: 67.1	Aerobic exercise	Stretching and relaxation	2	6 months	Overall cognitive function
		I: 65.3	Resistance exercise				
Newton et al. (2020)	46/48	C: 53.7	Aerobic exercise	Thematic education	2	5 months	Blood sugar, Lipid metabolis, Peak oxygen uptake
		I: 50.1	Resistance exercise				
Sénéchal et al. (2013)	31/60	C: 58.6	Aerobic exercise	Stretching and relaxation	2	9 months	Physical fitness
		I: 55.9	Resistance exercise				
Annibalini et al. (2017)	16/0	C: 60.0	Aerobic exercise	Routine care	3	16 weeks	HbA1c, Blood sugar, Lipid metabolis, Physical fitness
		I: 57.0	resistance exercise				
Donyaei et al. (2024)	0/34	C: 61.3	Aerobic exercise	No intervention	3	12 weeks	Blood sugar
		I: 62.1	Resistance exercise				
Larose et al. (2010)	81/46	C: 54.8	Aerobic exercise	Sedentary	3	6 months	Peak oxygen uptake
		I: 53.5	Resistance exercise				
Byrkjeland et al. (2015)	95/19	C: 63.2	Aerobic exercise	Routine medical follow-up	3	12 months	HbA1c, Peak oxygen uptake
		I: 64.6	Resistance exercise				
Banitalebi et al. (2019)	0/28	C: 55.71	Aerobic exercise	Routine care	3	10 weeks	HbA1c, Blood sugar, Physical fitness
		I: 54.14	Resistance exercise				
Sparks et al. (2013)	8/14	C: 60.8	Aerobic exercise	Stretching and relaxation	2	9 months	HbA1c, Physical fitness
		I: 54.1	Resistance exercise				
Banitalebi et al. (2021)	0/28	C: 55.71	Aerobic exercise	Routine care	3	10 weeks	HbA1c, Blood sugar, Lipid metabolis, BMI
		I: 54.14	Resistance exercise				
Kadoglou et al. (2013)	12/34	C: 57.9	Aerobic exercise	Physical activity	4	6 months	HbA1c, Blood sugar, Lipid metabolis, Physical fitness
		I: 57.9	Resistance exercise				
De Oliveira et al. (2012)	8/14	C: 53.42	Aerobic exercise	Sedentary	3	12 weeks	HbA1c, Blood sugar, Peak oxygen uptake
		I: 57.9	Resistance exercise				
Jamshidpour, Bahrpeyma & Khatami (2020)	20/8	C: 58.46	Aerobic exercise	No intervention	3	8 weeks	Quality of life
		I: 64.93	Resistance exercise				
Delevatti et al. (2022)	20/18	C: 58.6	Aquatic aerobic training	Stretching and relaxation	3	15 weeks	HbA1c, Blood sugar, Lipid metabolis
		I: 60.9	Aquatic resistance training				
Delevatti et al. (2020)	20/18	C: 58.6	Aquatic aerobic training	Stretching and relaxation	3	15 weeks	Peak oxygen uptake
		I: 60.9	Aquatic resistance training				
Kang, Ko & Baek (2016)	0/16	C: 57.5	Walking resistance exercise	Sedentary	3	12 weeks	Blood sugar, Lipid metabolis, Peak oxygen uptake
		I: 56.0					
Ghodrati et al. (2023)	0/21	C: 55.8	Aerobic exercise	Sedentary	3	12 months	HbA1c, Blood sugar, Physical fitness, Overall cognitive function
		I: 58.8	Resistance exercise				
			Balance exercise				
Martínez-Velilla et al. (2021)	50/53	C: 86.0	Aerobic exercise	Routine care	5–7	Continued for 8 days or until discharge	Overall cognitive function
		I: 87.0	Resistance exercise				
			Balance exercise				
Espeland et al. (2017)	155/260	70–89	Aerobic exercise	Health education	3–4	24 months	Overall cognitive function
			Resistance exercise				
			Flexibility and balance training				
Szilágyi et al. (2019)	74/134	C: 60.10	Aerobic exercise	No intervention	3–4	24 weeks	BMI
		I: 61.83	Resistance exercise				
			Recreation sports				
Jeon et al. (2020)	0/35	C:61.1	Folk dance	No intervention	3	12 weeks	HbA1c, Lipid metabolis, BMI
		I: 62.1	Resistance exercise				
Cai et al. (2023)	13/21	C: 61.82	Yi jin jing	No intervention	5	6 months	HbA1c, Blood sugar, Lipid metabolis, BMI
		I: 63.41	Resistance exercise				
Venkataraman et al. (2019)	63/80	62	Power training	Drug therapy	1	8 weeks	Quality of life
			Balance exercise				
Yue et al. (2023)	45/38	60.57	Tai Chi	health education	3	12 weeks	Quality of life
			Mindfulness training				
Ahmad et al. (2020)	25/13	C: 57.24	Sensorimotor	Routine care	3	8 weeks	Muscle function
		I: 60.33	Gait training				

**Note:**

I, Intervention group; C, Control group.

### Risk of bias assessment

**Figure 2:**
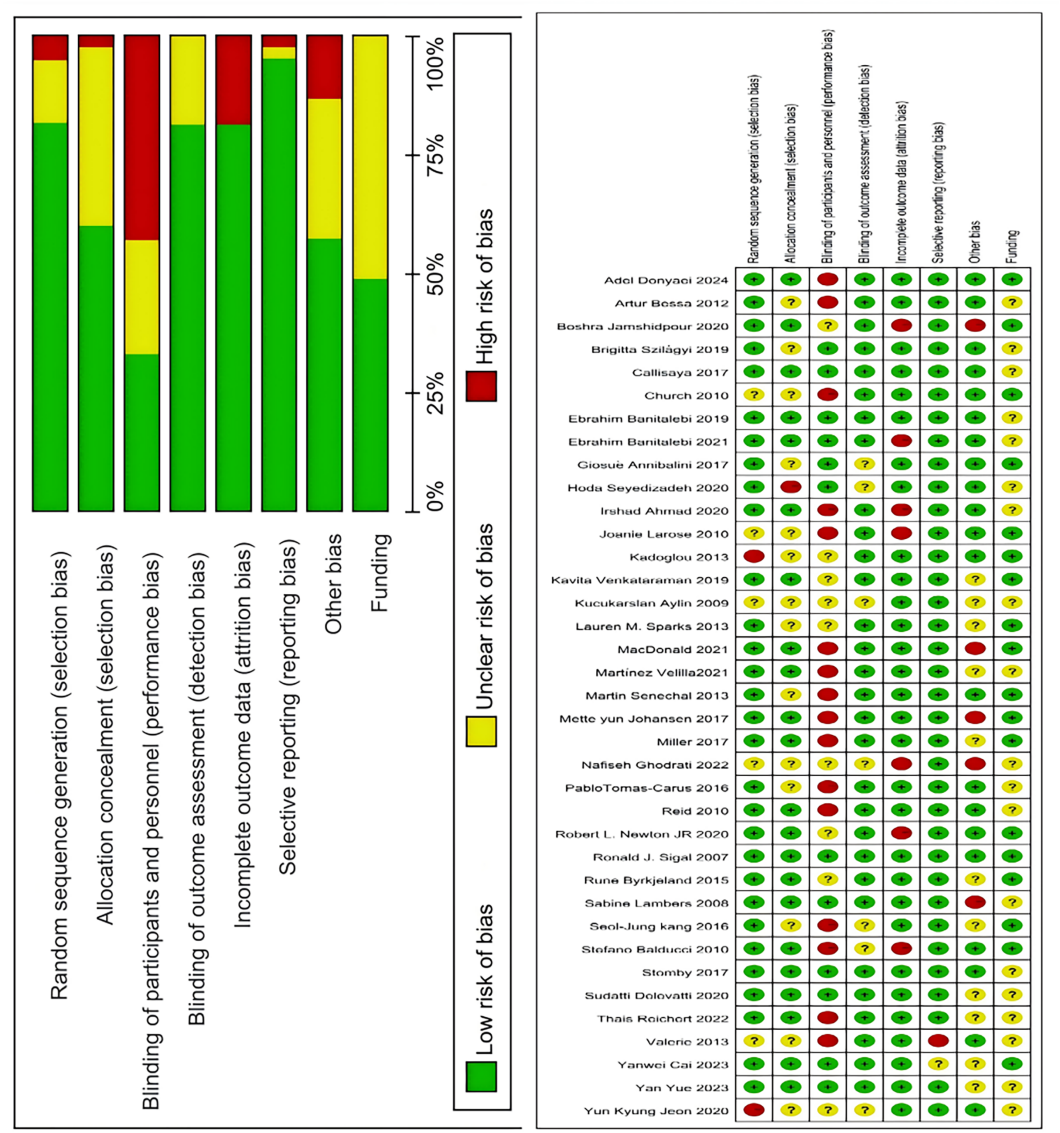
Analysis of the risk of bias in accordance with the cochrane collaboration guidelines (Sigal et al., 2007; Church et al., 2010; Lambers et al., 2008; Tomas-Carus et al., 2016; Seyedizadeh, Cheragh-Birjandi & Hamedi Nia, 2020; Johansen et al., 2017; Macdonald et al., 2021; Aylin et al., 2009; Myers et al., 2013; Balducci et al., 2010; Reid et al., 2010; Stomby et al., 2017; Callisaya et al., 2017; Newton et al., 2020; Sénéchal et al., 2013; Annibalini et al., 2017; Donyaei et al., 2024; Larose et al., 2010; Byrkjeland et al., 2015; Banitalebi et al., 2019; Sparks et al., 2013; Banitalebi et al., 2021; Kadoglou et al., 2013; De Oliveira et al., 2012; Jamshidpour, Bahrpeyma & Khatami, 2020; Delevatti et al., 2022, 2020; Kang, Ko & Baek, 2016; Ghodrati et al., 2023; Martínez-Velilla et al., 2021; Espeland et al., 2017; Szilágyi et al., 2019; Jeon et al., 2020; Cai et al., 2023; Venkataraman et al., 2019; Yue et al., 2023; Ahmad et al., 2020).

Two researchers independently assessed the quality of evidence for each outcome using Cochrane review standards. In cases of disagreement, a third researcher was consulted until consensus was achieved. According to pre-set quality assessment criteria shown in Fig. 2, 31 articles had five or more low-risk items, four articles had three to four low-risk items, and two articles had two or fewer low-risk items. The final assessment categorized 31 articles as Grade A, four as Grade B, and two as Grade C.

### Comprehensive results analysis

#### Analysis of multi-component exercise intervention on T2DM HbA1c and fasting glucose

**Figure 3:**
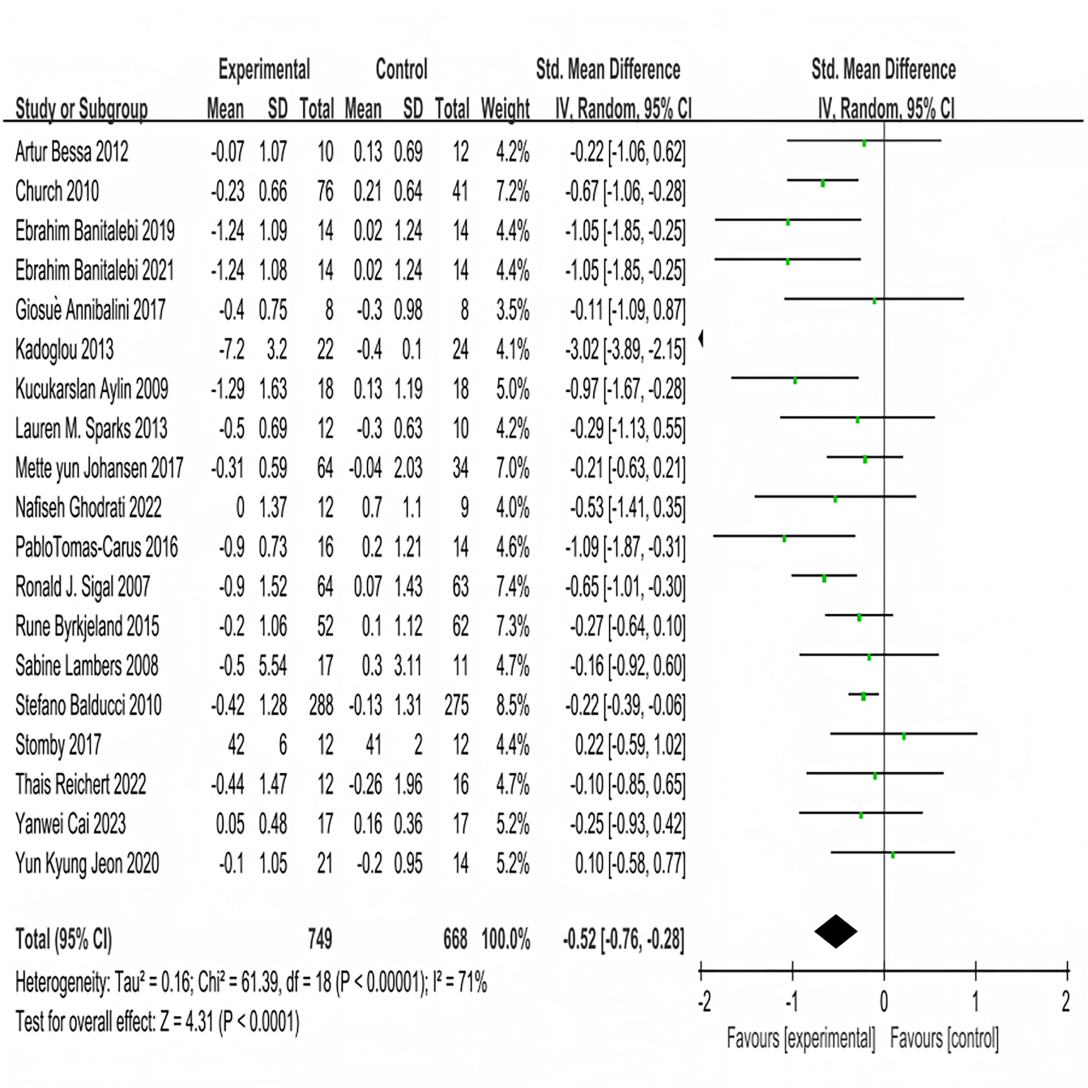
Forest plot of the effect of multi-component exercise on HbA1c in patients with type 2 diabetes mellitus (De Oliveira et al., 2012; Church et al., 2010; Banitalebi et al., 2019, 2021; Annibalini et al., 2017; Kadoglou et al., 2013; Aylin et al., 2009; Sparks et al., 2013; Johansen et al., 2017; Ghodrati et al., 2023; Tomas-Carus et al., 2016; Sigal et al., 2007; Byrkjeland et al., 2015; Lambers et al., 2008; Balducci et al., 2010; Stomby et al., 2017; Delevatti et al., 2022; Cai et al., 2023; Jeon et al., 2020).

Nineteen studies investigated the impact of multi-component exercise on HbA1c levels in patients with T2DM, involving a total of 1,417 participants (Fig. 3). Heterogeneity testing results indicated: χ^2^ = 61.39, *P* < 0.001, I^2^ = 71%, demonstrating heterogeneity among the studies. The pooled effect size was: SMD = −0.52, 95% CI [−0.76 to −0.28], *P* < 0.001 (Fig. 3). The forest plot showed that the 95% CI did not intersect with the line of no effect (Fig. 3). These results suggest that multi-component exercise effectively improves HbA1c in patients with T2DM.

**Figure 4:**
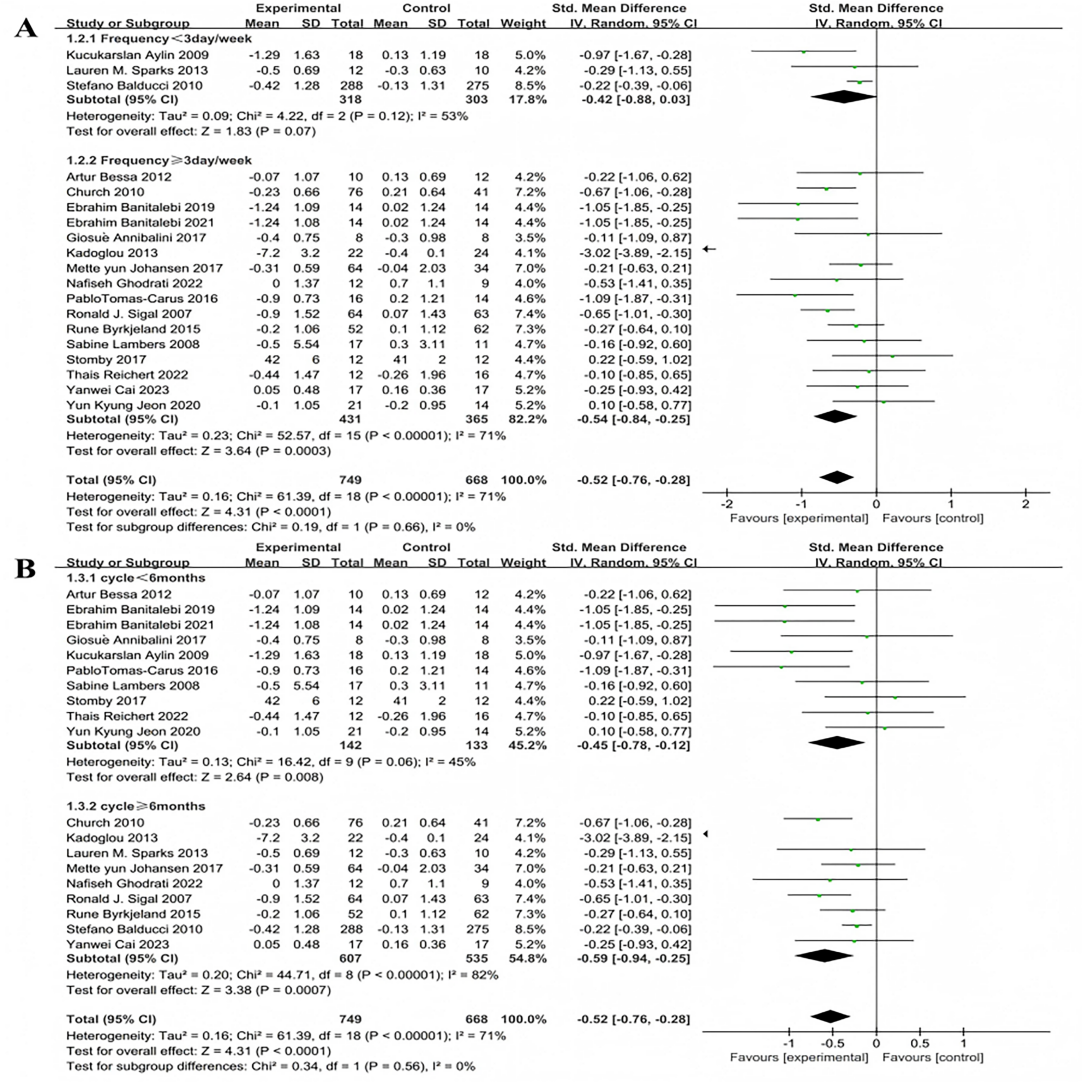
Forest plot of the effect of exercise-related regulation on HbA1c in patients with type 2 diabetes mellitus: (A) exercise frequency (B) exercise duration (De Oliveira et al., 2012; Church et al., 2010; Banitalebi et al., 2019, 2021; Annibalini et al., 2017; Kadoglou et al., 2013; Aylin et al., 2009; Sparks et al., 2013; Johansen et al., 2017; Ghodrati et al., 2023; Tomas-Carus et al., 2016; Sigal et al., 2007; Byrkjeland et al., 2015; Lambers et al., 2008; Balducci et al., 2010; Stomby et al., 2017; Delevatti et al., 2022; Cai et al., 2023; Jeon et al., 2020).

Subgroup analyses for exercise frequency were conducted on the 19 included studies. Exercise frequency was categorized into less than 3 days per week and 3 or more days per week (including 3 days). There was heterogeneity in effect size differences between these two groups (I^2^ = 71%), indicating that exercise frequency significantly modulates the intervention effects (Fig. 4A). Three studies investigated the effect of exercise less than 3 days per week on HbA1c improvement in patients with T2DM. The heterogeneity test results showed: χ^2^ = 4.22, I^2^ = 53%, *P* = 0.12, with a pooled effect size of SMD = −0.42, 95% CI [−0.88 to 0.03], *P* = 0.07, indicating no statistically significant difference. Sixteen studies examined the effect of exercising 3 or more days per week on HbA1c improvement in patients with T2DM. The heterogeneity test results showed: χ^2^ = 52.57, I^2^ = 71%, *P* < 0.001, with a pooled effect size of SMD = −0.54, 95% CI [−0.84 to −0.25], *P* = 0.0003, indicating a statistically significant difference.

Exercise duration was categorized into less than 6 months and 6 months or more (including 6 months). There was heterogeneity in effect size differences between these two groups (I^2^ = 71%), suggesting that exercise duration significantly modulates the intervention effects (Fig. 4B). Ten studies investigated the effect of exercise lasting less than 6 months on HbA1c improvement in patients with T2DM. The heterogeneity test results showed: χ^2^ = 16.42, I^2^ = 45%, *P* = 0.06, with a pooled effect size of SMD = −0.45, 95% CI [−0.78 to −0.12], *P* = 0.08, indicating a statistically significant difference. Nine studies examined the effect of exercise lasting 6 months or more on HbA1c improvement in patients with T2DM. The heterogeneity test results showed: χ^2^ = 44.71, I^2^ = 82%, *P* < 0.001, with a pooled effect size of SMD = −0.59, 95% CI [−0.94 to −0.25], *P* = 0.0007 indicating a statistically significant difference.

**Figure 5:**
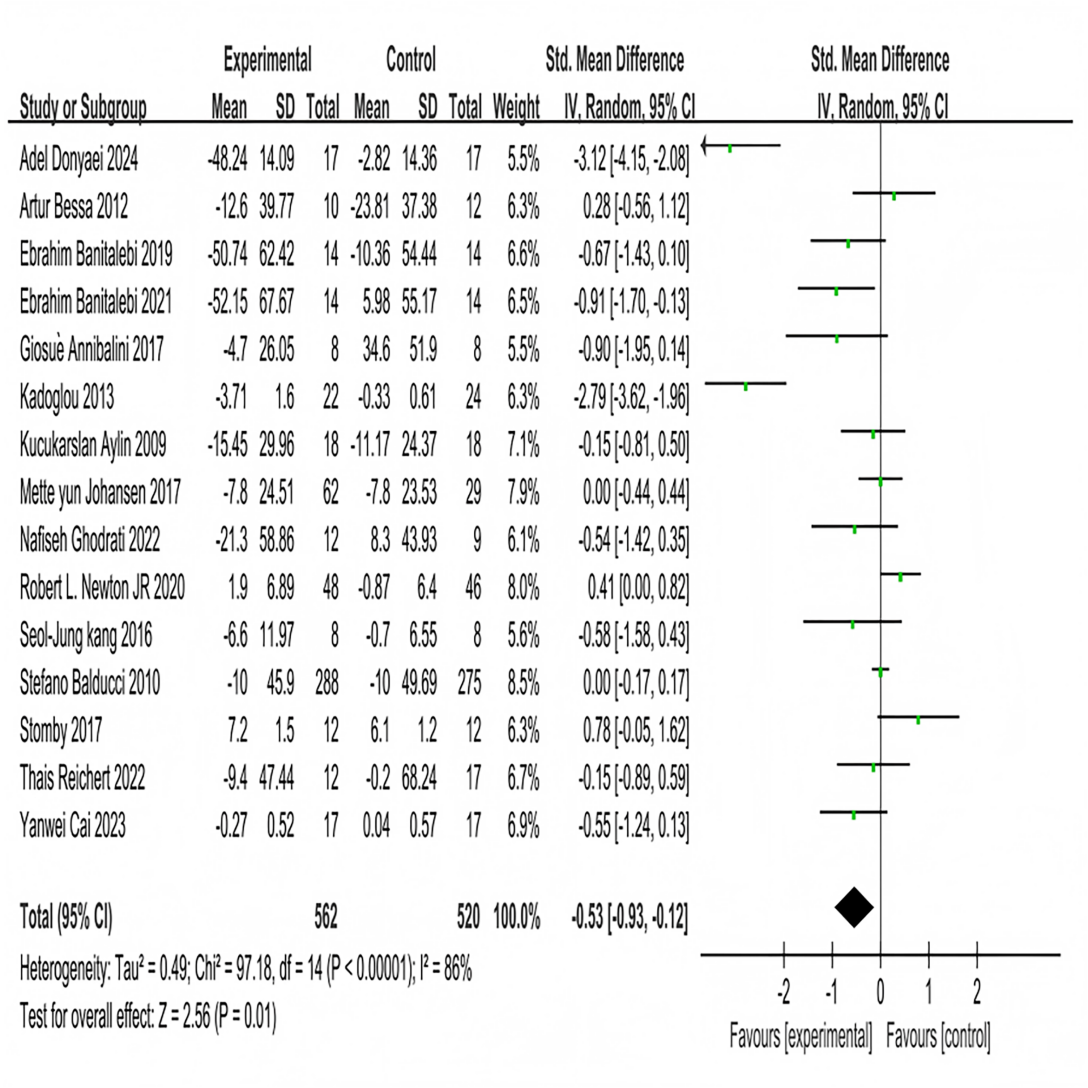
Forest plot of the effect of multi-component exercise on fasting blood glucose in patients with type 2 diabetes mellitus (Donyaei et al., 2024; De Oliveira et al., 2012; Banitalebi et al., 2019, 2021; Annibalini et al., 2017; Kadoglou et al., 2013; Aylin et al., 2009; Johansen et al., 2017; Ghodrati et al., 2023; Newton et al., 2020; Kang, Ko & Baek, 2016; Balducci et al., 2010; Stomby et al., 2017; Delevatti et al., 2022; Cai et al., 2023).

Fifteen studies investigated the effects of multi-component exercise on fasting blood glucose in patients with T2DM, involving a total of 1,080 participants (Fig. 5). The results of the heterogeneity test showed χ^2^ = 97.18, *P* < 0.001, I^2^ = 86%, indicating substantial heterogeneity among the studies. This high heterogeneity may be attributed to variations in the specific components of the multi-component exercise interventions (*e.g*., exercise frequency, duration), differences in participant characteristics (*e.g*., age range, baseline glycemic control), and other factors. The pooled effect size was SMD = −0.53, 95% CI [−0.93 to −0.12], *P* = 0.01. The forest plot demonstrated that the 95% CI did not intersect with the null line (Fig. 5). These results indicate a significant effect of multi-component exercise intervention on changes in fasting blood glucose levels in patients with T2DM.

**Figure 6:**
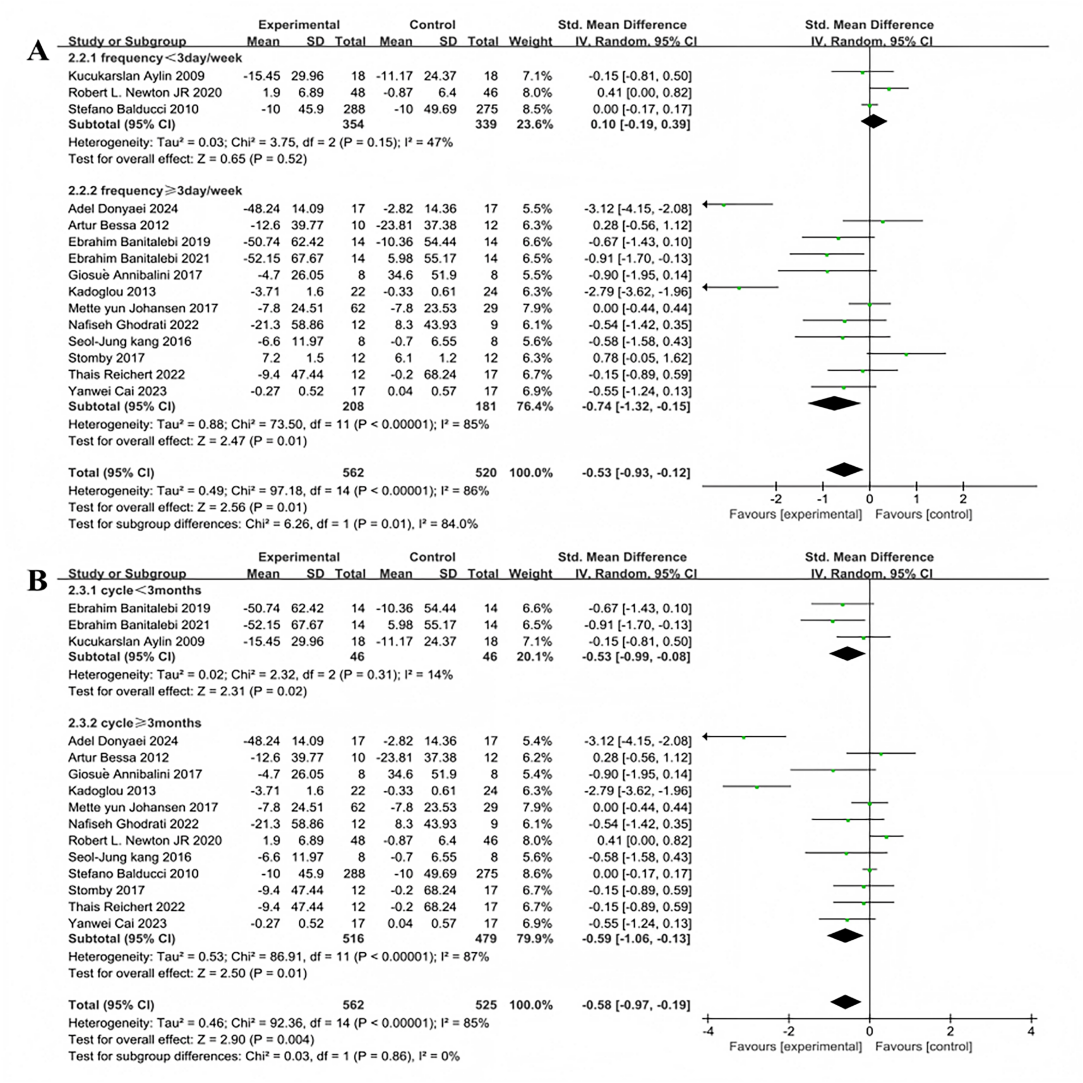
Forest plot of the effect of exercise-related regulation on fasting blood glucose in patients with type 2 diabetes mellitus: (A) exercise frequency (B) exercise duration (Donyaei et al., 2024; De Oliveira et al., 2012; Banitalebi et al., 2019, 2021; Annibalini et al., 2017; Kadoglou et al., 2013; Aylin et al., 2009; Johansen et al., 2017; Ghodrati et al., 2023; Newton et al., 2020; Kang, Ko & Baek, 2016; Balducci et al., 2010; Stomby et al., 2017; Delevatti et al., 2022; Cai et al., 2023).

Subgroup analyses were conducted on the included 15 studies. Exercise frequency was divided into groups of less than 3 days per week and 3 days or more per week (including 3 days), showing significant heterogeneity in effect sizes between the two groups (I^2^ = 84%), indicating a significant moderating effect of exercise frequency on intervention efficacy (Fig. 6A). Three studies investigated the effect of exercise less than 3 days per week on improving fasting blood glucose in T2DM patients. The heterogeneity test results showed χ^2^ = 3.75, I^2^ = 47%, *P* = 0.15, with a pooled effect size of SMD = 0.10, 95% CI [−0.19 to 0.39], *P* = 0.52, indicating no statistically significant difference. Twelve studies examined the effect of exercising 3 or more days per week on improving fasting blood glucose in T2DM patients. The heterogeneity test results showed χ^2^ = 73.5, I^2^ = 85%, *P* < 0.001, with a pooled effect size of SMD = −0.74, 95% CI [−1.32 to −0.15], *P* = 0.01, indicating a statistically significant difference. Exercise duration was categorized into less than 3 months and 3 months or more (including 3 months), with significant heterogeneity in effect size differences between the two groups (I^2^ = 85%), indicating that exercise duration significantly moderates the intervention effects (Fig. 6B). Three studies investigated the effect of exercise lasting less than 3 months on improving fasting blood glucose in patients with T2DM. The heterogeneity test results showed: χ^2^ = 2.32, I^2^ = 14%, *P* = 0.31, with a pooled effect size of SMD = −0.53, 95% CI [−0.99 to −0.08], P = 0.02, indicating a statistically significant difference. Twelve studies examined the effect of exercise lasting 3 months or more on improving fasting blood glucose in patients with T2DM. The heterogeneity test results showed: χ^2^ = 86.91, I^2^ = 87%, *P* < 0.001, with a pooled effect size of SMD = −0.59, 95% CI [−1.06 to −0.13], *P* = 0.01, indicating a statistically significant difference.

#### Analysis of multi-component exercise intervention on T2DM lipid metabolism

**Figure 7:**
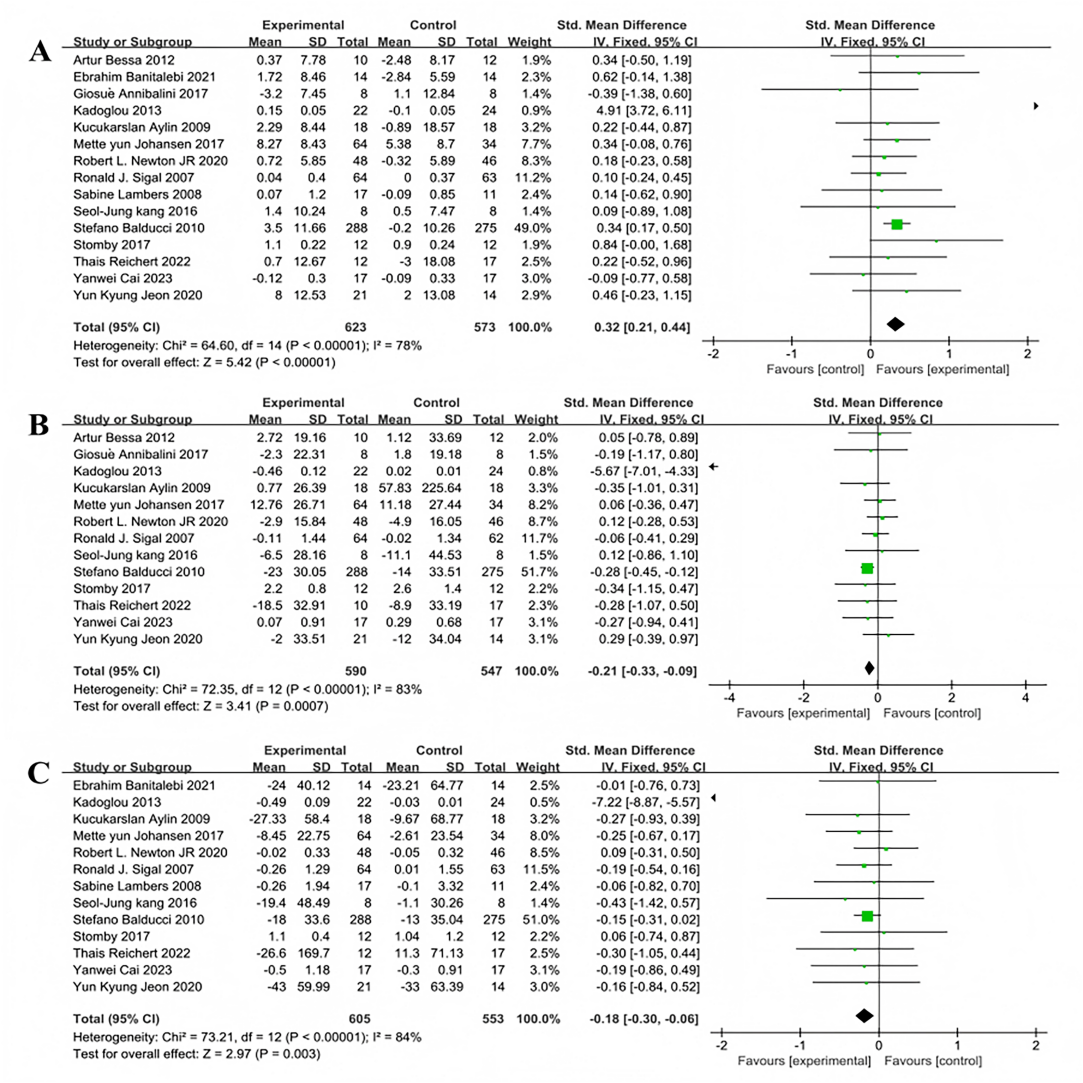
Forest plot of the effect of multi-component exercise on lipid metabolism in patients with type 2 diabetes mellitus: (A) high-density lipoprotein (De Oliveira et al., 2012; Banitalebi et al., 2021; Annibalini et al., 2017; Kadoglou et al., 2013; Aylin et al., 2009; Johansen et al., 2017; Newton et al., 2020; Sigal et al., 2007; Lambers et al., 2008; Kang, Ko & Baek, 2016; Balducci et al., 2010; Stomby et al., 2017; Delevatti et al., 2022; Cai et al., 2023; Jeon et al., 2020); (B) low-density lipoprotein (De Oliveira et al., 2012; Annibalini et al., 2017; Kadoglou et al., 2013; Aylin et al., 2009; Johansen et al., 2017; Newton et al., 2020; Sigal et al., 2007; Kang, Ko & Baek, 2016; Balducci et al., 2010; Stomby et al., 2017; Delevatti et al., 2022; Cai et al., 2023; Jeon et al., 2020); (C) triglyceride (Banitalebi et al., 2021; Kadoglou et al., 2013; Aylin et al., 2009; Johansen et al., 2017; Newton et al., 2020; Sigal et al., 2007; Lambers et al., 2008; Kang, Ko & Baek, 2016; Balducci et al., 2010; Stomby et al., 2017; Delevatti et al., 2022; Cai et al., 2023; Jeon et al., 2020).

Fifteen studies encompassing 1,196 participants assessed the effects of multi-component exercise on high density lipoprotein (HDL) levels in individuals with T2DM (Fig. 7A). Significant heterogeneity was noted among the studies (χ^2^ = 64.6, *P* < 0.001, I^2^ = 78%), and the pooled effect size was substantial (SMD = 0.32, 95% CI [0.21–0.44], *P* < 0.001) (Fig. 7A). The forest plot demonstrated that the 95% CI did not intersect with the line of no effect (Fig. 7A), indicating that multi-component exercise interventions effectively improve HDL levels in patients with T2DM. Thirteen studies involving 1,137 participants evaluated the impact of multi-component exercise on low density lipoprotein (LDL) levels ((Fig. 7B). Considerable heterogeneity was observed (χ^2^ = 72.35, *P* < 0.001, I^2^ = 83%), with a significant negative pooled effect size (SMD = −0.21, 95% CI [−0.33 to −0.09], *P* = 0.0007) ((Fig. 7B). The forest plot indicated that the 95% CI did not intersect with the line of no effect (Fig. 7B), suggesting that multi-component exercise has a significant impact on reducing LDL levels in patients with T2DM. Additionally, thirteen studies involving 1,158 participants investigated the effects of multi-component exercise on TG levels (Fig. 7C). High heterogeneity was also evident (χ^2^ = 73.21, *P* < 0.001, I^2^ = 84%), and the pooled effect size indicated a significant improvement (SMD = −0.18, 95% CI [−0.3 to −0.06], *P* = 0.003) (Fig. 7C). The forest plot showed that the 95% CI did not intersect with the line of no effect (Fig. 7C), confirming that multi-component exercise interventions significantly improve TG levels in patients with T2DM.

**Figure 8:**
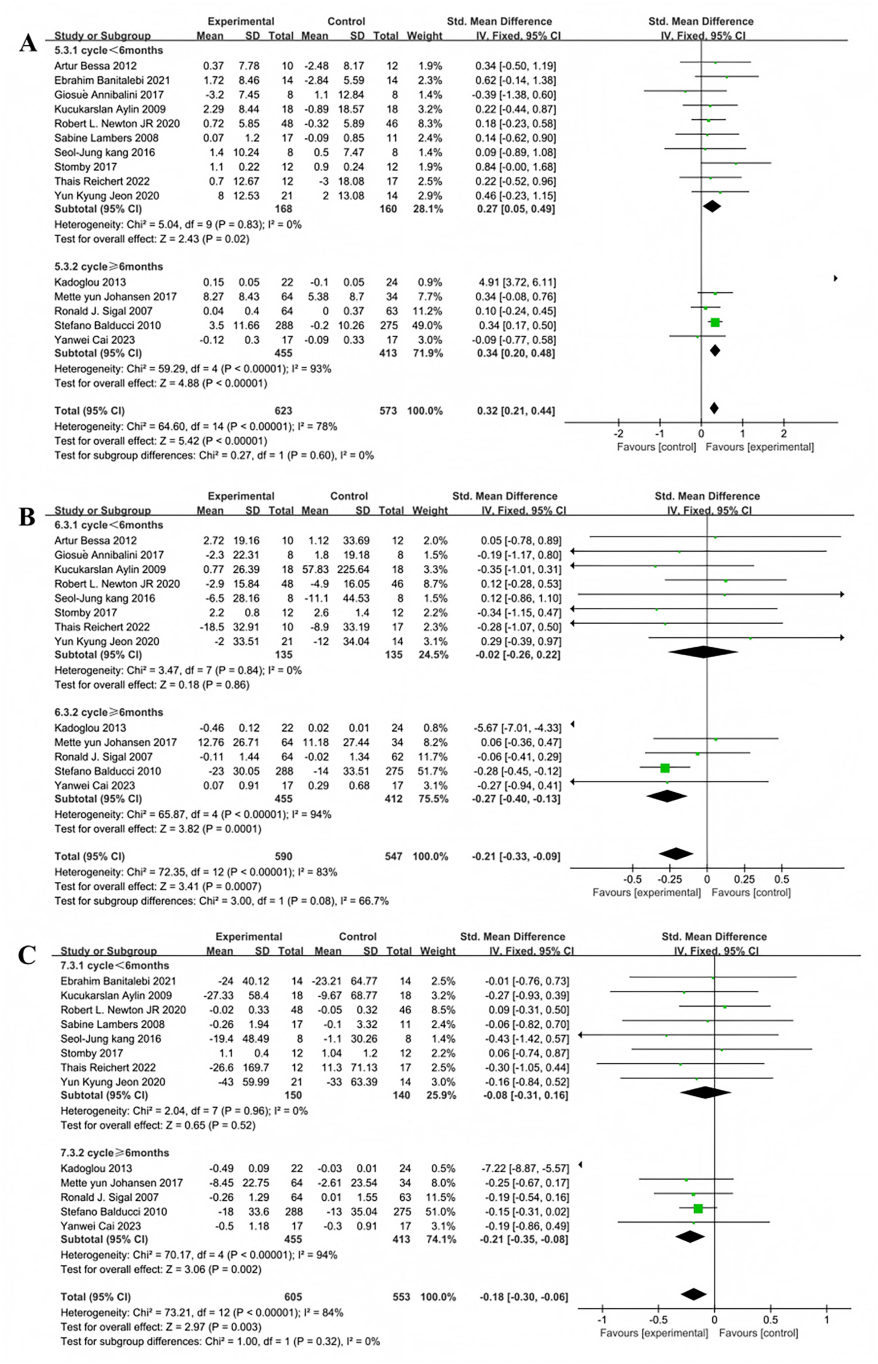
Forest plot of exercise duration regulation on lipid metabolism in patients with type 2 diabetes mellitus: (A) High density lipoprotein-exercise duration (De Oliveira et al., 2012; Banitalebi et al., 2021; Annibalini et al., 2017; Kadoglou et al., 2013; Aylin et al., 2009; Johansen et al., 2017; Newton et al., 2020; Sigal et al., 2007; Lambers et al., 2008; Kang, Ko & Baek, 2016; Balducci et al., 2010; Stomby et al., 2017; Delevatti et al., 2022; Cai et al., 2023; Jeon et al., 2020); (B) Low density lipoprotein-exercise duration (De Oliveira et al., 2012; Annibalini et al., 2017; Kadoglou et al., 2013; Aylin et al., 2009; Johansen et al., 2017; Newton et al., 2020; Sigal et al., 2007; Kang, Ko & Baek, 2016; Balducci et al., 2010; Stomby et al., 2017; Delevatti et al., 2022; Cai et al., 2023; Jeon et al., 2020); (C) Triglyceride-exercise duration (Banitalebi et al., 2021; Kadoglou et al., 2013; Aylin et al., 2009; Johansen et al., 2017; Newton et al., 2020; Sigal et al., 2007; Lambers et al., 2008; Kang, Ko & Baek, 2016; Balducci et al., 2010; Stomby et al., 2017; Delevatti et al., 2022; Cai et al., 2023; Jeon et al., 2020).

Subgroup analyses of the 15 studies evaluating the effect of multi-component exercise on HDL levels were stratified by exercise duration into less than 6 months and 6 months or more, revealing significant heterogeneity between the groups (I^2^ = 78%), indicating a substantial moderating effect of exercise duration on intervention outcomes (Fig. 8A). For exercise durations less than 6 months,the heterogeneity test showed no variability (χ^2^ = 5.04, I^2^ = 0%, *P* = 0.83) and a significant pooled effect size (SMD = 0.27, 95% CI [0.05–0.49], *P* = 0.02). For durations of 6 months or more, substantial heterogeneity was observed (χ^2^ = 59.29, I^2^ = 93%, *P* < 0.001) with a significant pooled effect size (SMD = 0.34, 95% CI [0.20–0.48], *P* < 0.001).

Subgroup analyses of the 13 studies that included LDL as an outcome were also divided by exercise duration, showing significant heterogeneity (I^2^ = 83%) and indicating that duration significantly modulates the effects of exercise interventions (Fig. 8B). For durations less than 6 months, heterogeneity was negligible (χ^2^ = 3.47, I^2^ = 0%, *P* = 0.84) with a non-significant pooled effect size (SMD = −0.02, 95% CI [−0.26 to 0.22], *P* = 0.86). For durations of 6 months or more, very high heterogeneity was present (χ^2^ = 65.87, I^2^ = 94%, *P* < 0.001) with a significant pooled effect size (SMD = −0.27, 95% CI [−0.40 to −0.13], *P* = 0.0001).

Finally, subgroup analyses for the 13 studies assessing the impact of exercise on TG were categorized by duration, showing significant heterogeneity (I^2^ = 84%) and indicating a significant moderating effect of exercise duration (Fig. 8C). For exercise periods less than 6 months, no significant heterogeneity was observed (χ^2^ = 2.04, I^2^ = 0%, *P* = 0.96), and the pooled effect size was non-significant (SMD = −0.08, 95% CI [−0.31 to 0.16], *P* = 0.52). For periods of 6 months or more, very high heterogeneity was evident (χ^2^ = 70.17, I^2^ = 94%, *P* < 0.001) with a significant pooled effect size (SMD = −0.21, 95% CI [−0.35 to −0.08], *P* = 0.002).

#### Analysis of multi-component exercise intervention on T2DM physical fitness

**Figure 9:**
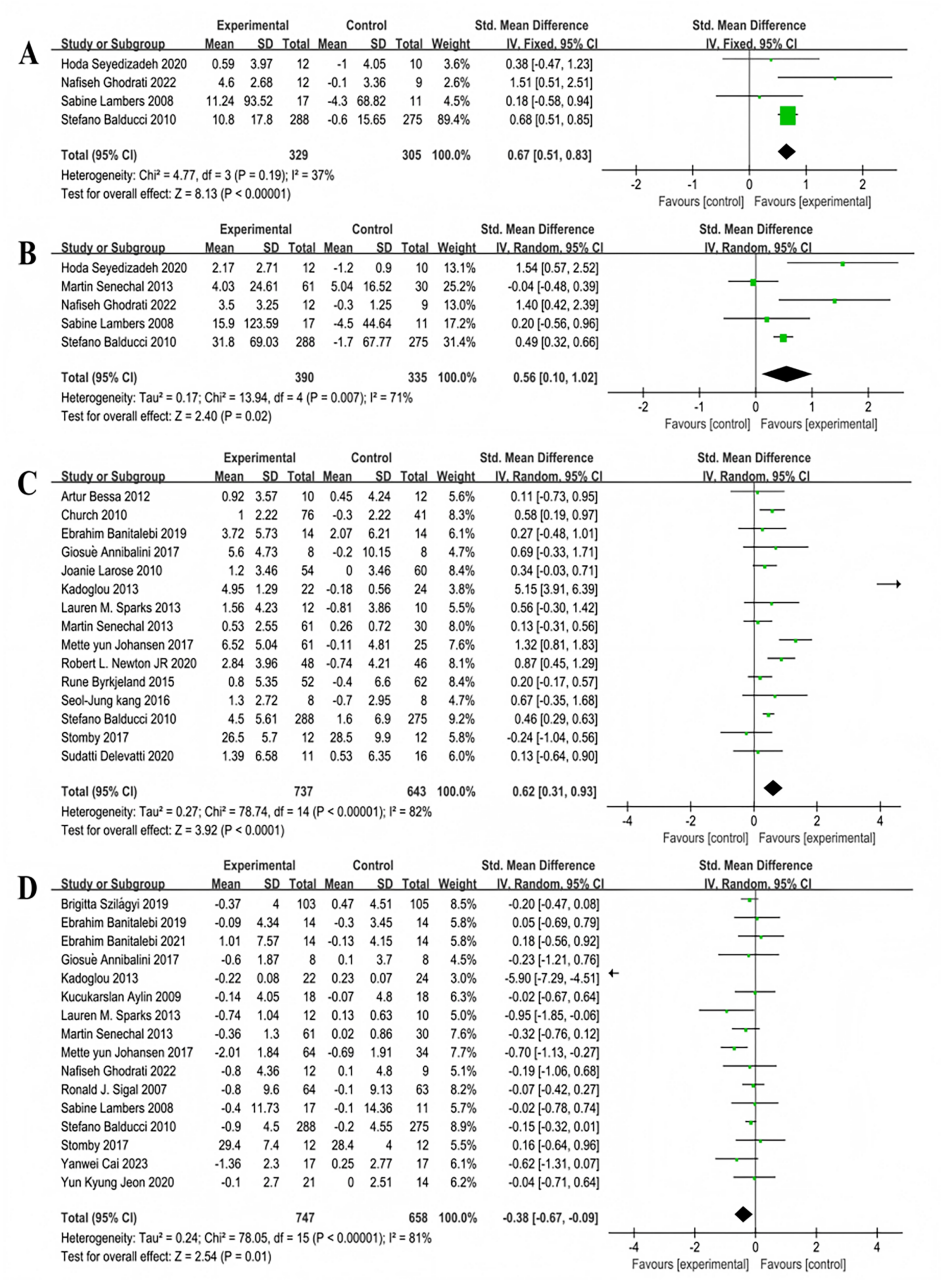
Forest plot of the effect of multi-component exercise on physical fitness in patients with type 2 diabetes mellitus: (A) upper limb strength (Seyedizadeh, Cheragh-Birjandi & Hamedi Nia, 2020; Ghodrati et al., 2023; Lambers et al., 2008; Balducci et al., 2010); (B) lower limb strength (Seyedizadeh, Cheragh-Birjandi & Hamedi Nia, 2020; Sénéchal et al., 2013; Ghodrati et al., 2023; Lambers et al., 2008; Balducci et al., 2010); (C) peak relative oxygen uptake (De Oliveira et al., 2012; Church et al., 2010; Annibalini et al., 2017; Banitalebi et al., 2019; Larose et al., 2010; Kadoglou et al., 2013; Sparks et al., 2013; Sénéchal et al., 2013; Johansen et al., 2017; Newton et al., 2020; Byrkjeland et al., 2015; Kang, Ko & Baek, 2016; Balducci et al., 2010; Stomby et al., 2017; Delevatti et al., 2022); (D) body mass index (BMI) (Szilágyi et al., 2019; Banitalebi et al., 2019; Banitalebi et al., 2021; Annibalini et al., 2017; Kadoglou et al., 2013; Aylin et al., 2009; Sparks et al., 2013; Sénéchal et al., 2013; Johansen et al., 2017; Ghodrati et al., 2023; Sigal et al., 2007; Lambers et al., 2008; Balducci et al., 2010; Stomby et al., 2017; Cai et al., 2023; Jeon et al., 2020).

Four studies reported the effects of multi-component exercise interventions on upper limb strength in individuals with T2DM, with a total of 634 participants (Fig. 9A). Heterogeneity test results: χ^2^ = 4.77, *P* = 0.19, I^2^ = 37%, indicating no significant heterogeneity among the studies. The pooled effect size was: SMD = 0.67, 95% CI [0.51–0.83], *P* < 0.001 (Fig. 9A). The forest plot showed: the 95% CI did not intersect with the line of no effect (Fig. 9A). Five studies reported the effects of multi-component exercise on lower limb strength in patients with T2DM, with a total of 725 participants (Fig. 9B). Heterogeneity test results: χ^2^ = 13.94, *P* = 0.007, I^2^ = 71%, indicating heterogeneity among the studies. The pooled effect size was: SMD = 0.56, 95% CI [0.10–1.02], *P* = 0.02 (Fig. 9B). The forest plot showed: the 95% CI did not intersect with the line of no effect (Fig. 9B). The results indicate that multi-component exercise significantly improves both upper and lower limb muscle strength in patients with T2DM.

Fifteen studies reported the effects of multi-component exercise interventions on peak oxygen uptake (VO2peak) in patients with T2DM, involving a total of 1,380 participants (Fig. 9C). Heterogeneity test results: χ^2^ = 78.74, *P* < 0.001, I^2^ = 82%, indicating significant heterogeneity among the studies. The pooled effect size was: SMD = 0.62, 95% CI [0.31–0.93], *P* < 0.001 (Fig. 9C). The forest plot showed: the 95% CI did not intersect with the line of no effect (Fig. 9C). The results indicate that multi-component exercise significantly increases the level of VO2peak in patients with T2DM.

Sixteen studies reported the effects of multi-component exercise interventions on BMI in patients with T2DM, involving a total of 1,405 participants (Fig. 9D). Heterogeneity test results: χ^2^ = 78.05, *P* < 0.001, I^2^ = 81%, indicating heterogeneity among the 16 studies on BMI. The pooled effect size was: SMD = −0.38, 95% CI [−0.67 to −0.09], *P* = 0.01 (Fig. 9D). The forest plot showed: the 95% CI did not intersect with the line of no effect (Fig. 9D). The results indicate that multi-component exercise significantly reduces the BMI index in patients with T2DM.

#### Analysis of multi-component exercise intervention on overall cognitive function in patients with T2DM

**Figure 10:**
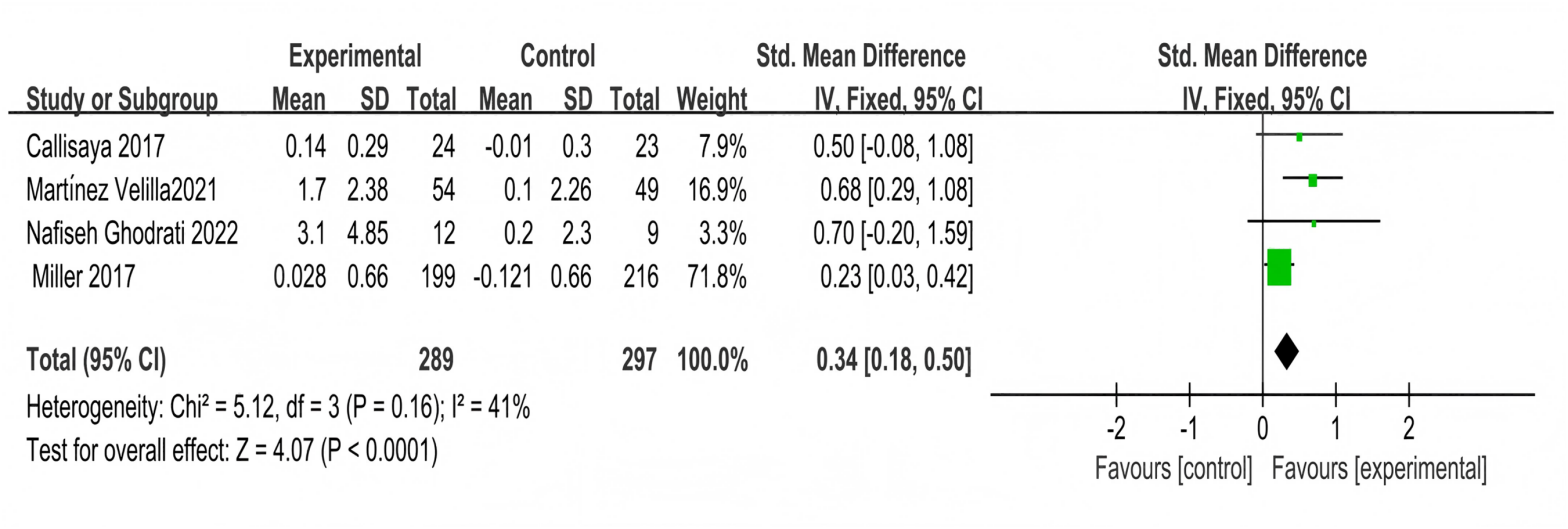
Forest plot of the effect of multi-component exercise on overall cognitive function in patients with type 2 diabetes mellitus (Callisaya et al., 2017; Martínez-Velilla et al., 2021; Ghodrati et al., 2023; Espeland et al., 2017).

Four studies involving 586 participants explored the impact on overall cognitive function,showing moderate heterogeneity (χ^2^ = 5.12, *P* = 0.1, I^2^ = 41%), and a combined effect size of SMD = 0.34, 95% CI [0.18–0.50], *P* < 0.001 (Fig. 10), with the forest plot indicating a significant effect (Fig. 10).

#### Analysis of the impact of multi-component exercise on the quality of life in patients with T2DM

**Figure 11:**
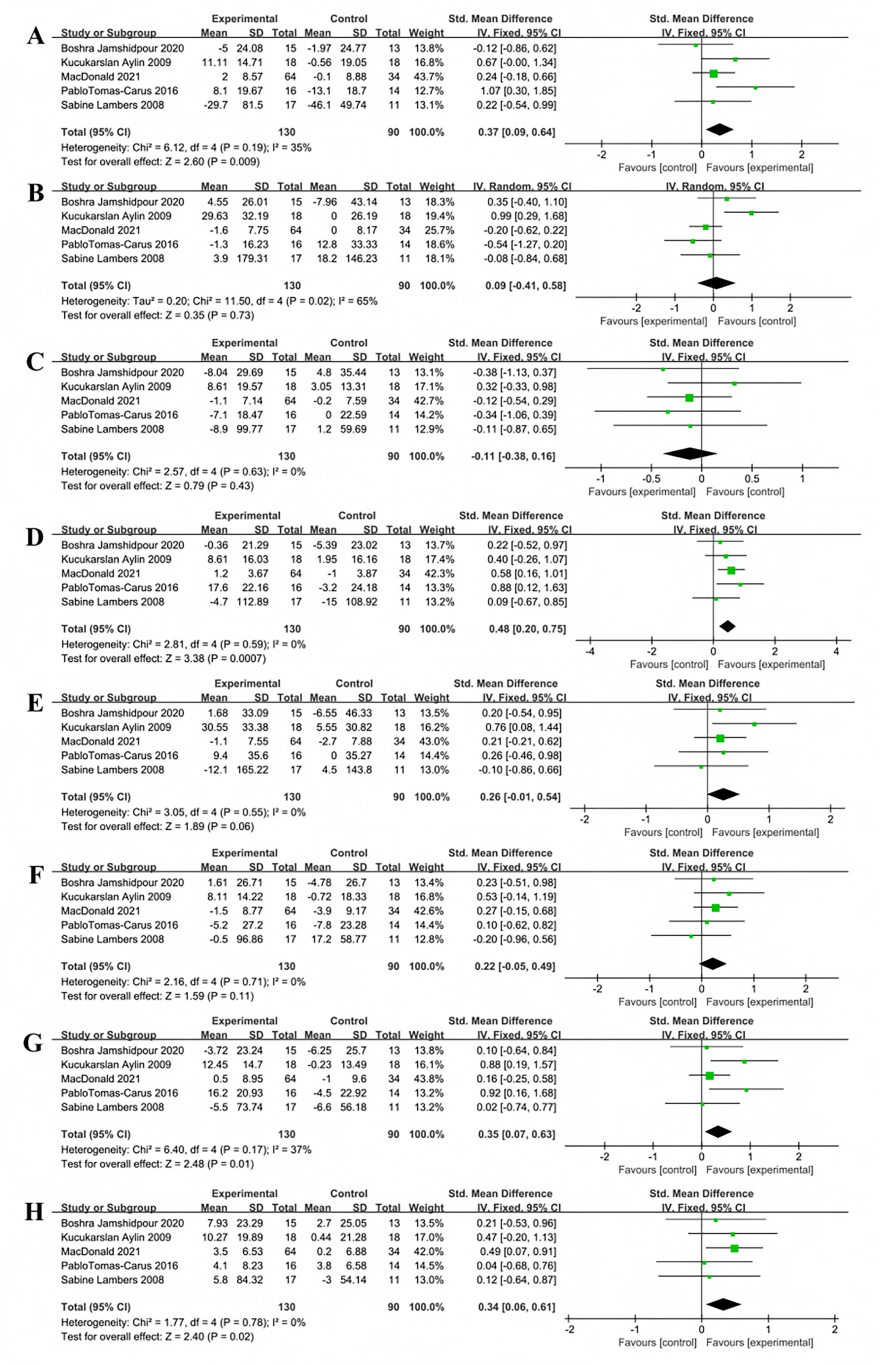
Forest plot of the effect of multi-component exercise on the quality of Life of patients with T2DM: (A) vitality; (B) emotional role functioning; (C) social functioning; (D) physical functioning; (E) physical role functioning; (F) bodily pain; (G) mental health; (H) general health (Jamshidpour, Bahrpeyma & Khatami, 2020; Aylin et al., 2009; Macdonald et al., 2021; Tomas-Carus et al., 2016; Lambers et al., 2008).

Five studies used the SF-36 scale to assess the impact of multi-component exercise on the quality of life in patients with T2DM, with a total of 220 participants. In terms of vitality, no significant heterogeneity was observed between the studies (X^2^ = 6.12, *P* = 0.19, I^2^ = 35%). The combined results from the forest plot (Fig. 11A) indicated that multi-component exercise significantly improved vitality in patients with T2DM (SMD = 0.37, 95% CI [0.09–0.64], *P* = 0.009). Regarding emotional role functioning, the combined results from the forest plot (Fig. 11B) showed that multi-component exercise had no significant effect on emotional role functioning in patients with T2DM (SMD = 0.09, 95% CI [−0.41 to 0.58], *P* = 0.73), with significant heterogeneity between the studies (X^2^ = 11.50, *P* = 0.02, I^2^ = 65%).

For social functioning, the combined results from the forest plot (Fig. 11C) showed that multi-component exercise did not significantly improve social functioning in patients with T2DM (SMD = −0.11, 95% CI [−0.38 to 0.16], *P* = 0.43), with no significant heterogeneity between the studies (X^2^ = 2.57, *P* = 0.63, I^2^ = 0%). In terms of physical functioning, the combined results from the forest plot (Fig. 11D) indicated that multi-component exercise effectively improved physical functioning patients with T2DM (SMD = 0.48, 95% CI [0.20–0.75], *P* = 0.007), with no significant heterogeneity between the studies (X^2^ = 2.81, *P* = 0.59, I^2^ = 0%).

For physical role functioning, the forest plot results (Fig. 11E) showed that multi-component exercise had no significant effect on physical role functioning in patients with T2DM (SMD = 0.26, 95% CI [−0.01 to 0.54], *P* = 0.06), with no significant heterogeneity between the studies (X^2^ = 3.05, *P* = 0.55, I^2^ = 0%). In terms of bodily pain, the combined results from the forest plot (Fig. 11F) showed that multi-component exercise did not significantly affect bodily pain in patients with T2DM (SMD = 0.22, 95% CI [−0.05 to 0.49], *P* = 0.11), with no significant heterogeneity between the studies (X^2^ = 2.16, *P* = 0.71, I^2^ = 0%).

Regarding mental health, the combined results from the forest plot (Fig. 11G) indicated that multi-component exercise significantly improved mental health levels in patients with T2DM (SMD = 0.35, 95% CI [0.07–0.63], *P* = 0.01), with no significant heterogeneity between the studies (X^2^ = 6.40, *P* = 0.17, I*2* = 37%). For general health perception, the combined results from the forest plot (Fig. 11H) showed that multi-component exercise effectively improved general health perception in patients with T2DM (SMD = 0.34, 95% CI [0.06–0.61], *P* = 0.02), with no significant heterogeneity between the studies (X^2^ = 1.77, *P* = 0.78, I^2^ = 0%).

### Sensitivity analysis and publication bias

**Figure 12:**
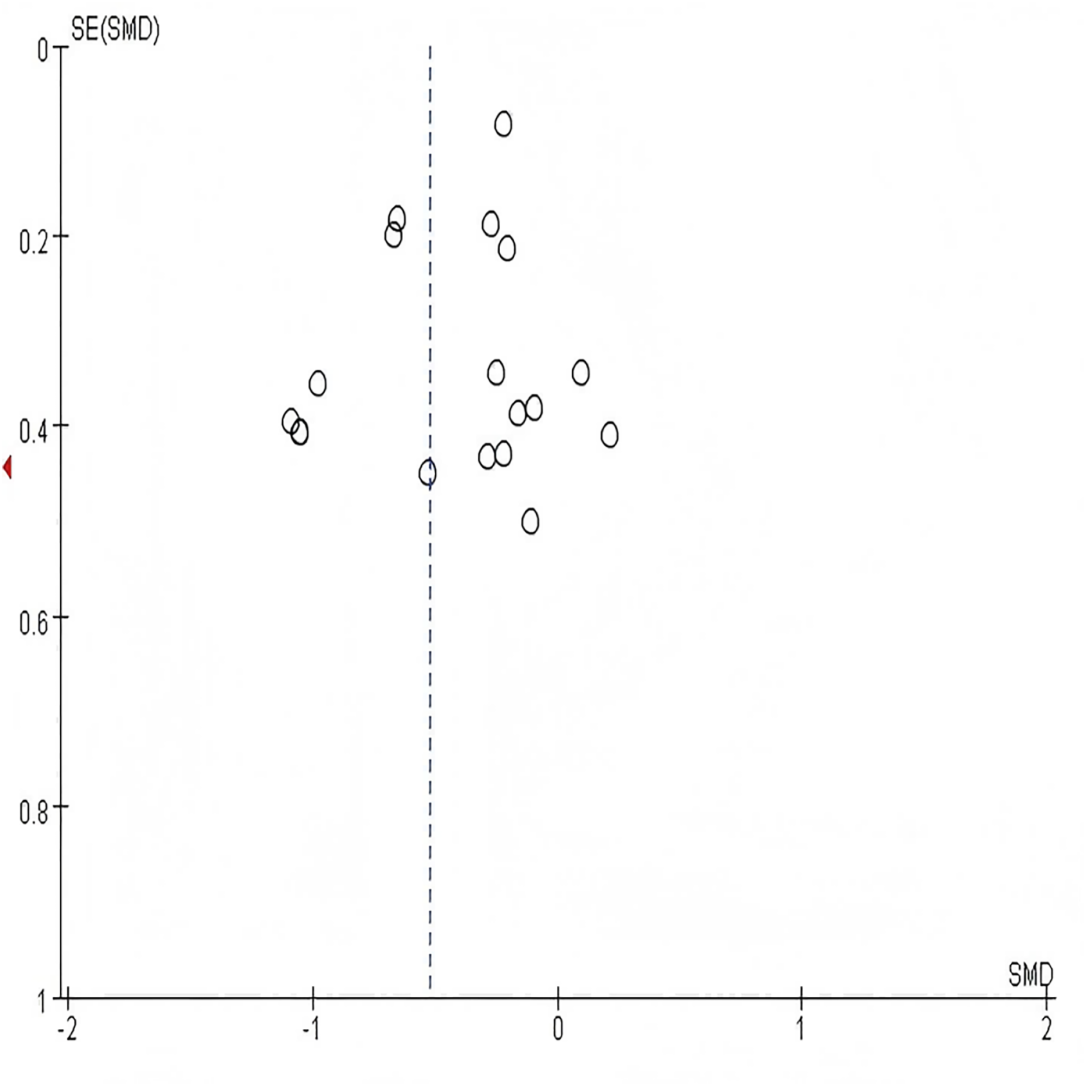
Funnel plot for HbA1c. The symmetrical distribution of studies around the pooled effect estimate (dashed vertical line) suggests an absence of significant publication bias.

In sensitivity analysis,the combined results of random-effects meta-analysis were consistent with those of fixed-effects meta-analysis or when studies with the lowest quality assessment score were excluded, indicating the stability of the pooled effects (SMD) of multi-component exercise intervention on HbA1c, blood glucose, metabolic health,cognitive function, body composition, physical function, and quality of life. Due to the complexity of the included study outcomes, funnel plots and analyses are typically conducted for meta-analyses involving more than 10 studies to explore publication bias. As shown in Fig. 12, all included studies exhibited a relatively symmetrical distribution. Subsequently, Egger’s test was performed for the study outcome indicator HbA1c, yielding *P* = 0.137 > 0.05 (Table 3), indicating no significant publication bias. Only the examination results for HbA1c are presented here; detailed results for other study outcome indicators are provided in Tables 4 through 9.

**Table 3:** Egger test results for HbA1c.

Std_Eff	Cofe	Std.Eff	*t*	*p* > |*t*|	(95% Conf. Interval)
Slope	−0.1521911	0.1859967	−0.82	0.425	[−0.54461 to 0.24023]
Bias	−1.215678	0.7785508	−1.56	0.137	[−2.85828 to 0.42692]

**Table 4:** Egger test results for fasting blood glucose.

Std_Eff	Cofe	Std.Eff	*t*	*p* > |*t|*	(95% Conf. Interval)
Slope	0.2858861	0.2578351	1.11	0.288	[−0.27113 to 0.84291]
Bias	−2.216822	1.058392	−2.09	0.056	[−4.50334 to 0.69694]

**Table 5:** Egger test results for high density lipoprotein.

Std_Eff	Cofe	Std.Eff	*t*	*p* > |*t*|	(95% Conf. Interval)
Slope	0.1566008	0.2329509	0.67	0.513	[−0.34666 to 0.65986]
Bias	0.8918643	1.011846	0.88	0.394	[−1.29409 to 3.07782]

**Table 6:** Egger test results for low density lipoprotein.

Std_Eff	Cofe	Std.Eff	*t*	*p* > |*t*|	(95% Conf. Interval)
Slope	−0.0539419	0.2712884	−0.20	0.846	[−0.65104 to 0.54316]
Bias	−0.8654674	1.235584	−0.70	0.498	[−3.58497 to 1.85403]

**Table 7:** Egger test results for triglyceride.

Std_Eff	Cofe	Std.Eff	*t*	*p* > |t|	(95% Conf. Interval)
Slope	0.1187733	0.2582417	0.46	0.655	[−0.44961 to 0.68716]
Bias	−1.674407	1.187914	−1.41	0.186	[−4.28899 to 0.94017]

**Table 8:** Egger test results for peak relative oxygen uptake.

Std_Eff	Cofe	Std.Eff	*t*	*p* > |t|	(95% Conf. Interval)
Slope	0.2877757	0.2536833	1.13	0.277	[−0.26027 to 0.83582]
Bias	1.165016	1.162796	1.00	0.335	[−1.34705 to 3.67708]

**Table 9:** Egger test results for body mass index.

Std_Eff	Cofe	Std.Eff	*t*	*p* > |*t*|	(95% Conf. Interval)
Slope	0.0188324	0.2237525	0.08	0.934	[−0.46107 to 0.49873]
Bias	−1.379875	1.020133	−1.35	0.198	[−3.56784 to 0.80809]

## Discussion

### Analysis of the overall effect of multi-component exercise on T2DM HbA1c and lipid metabolism

In patients with T2DM, as much as 50% to 80% of mortality is attributed to cardiovascular diseases, a proportion significantly higher than in people not living with T2DM. Studies indicate that long-term maintenance of elevated HbA1c levels is one of the key factors contributing to the development of cardiovascular diseases (Moliner-Urdiales et al., 2013; Hall et al., 2020). Additionally, a decrease in HbA1c levels is significantly associated with a reduced risk of mortality inpatients with T2DM (Wu et al., 2021). Furthermore, exercise is widely acknowledged to effectively reduce the risk of cardiovascular diseases in patients with T2DM. Multi-component exercise, as one of the non-pharmacological approaches to reducing or preventing the risk of mortality in T2DM, has been investigated for its differences with pharmacological treatments. Umpierrez et al. (2014) conducted a 52-week pharmacological treatment for T2DM, reporting that multi-component exercise had similar efficacy to metformin in reducing HbA1c levels. This meta-analysis demonstrates a significant reduction in HbA1c levels with multi-component exercise. To explore the differences between multi-component exercise and single exercise modalities in improving HbA1c levels in patients with T2DM, Church’s randomized controlled trial (Church et al., 2010) compared the effects of multi-component exercise and single exercises (resistance exercise, aerobic exercise) on HbA1c with the same weekly exercise time. This controlled for the impact of time differences on outcomes, showing that multi-component exercise had a 0.19% greater reduction in HbA1c compared to resistance exercise and a 0.11% greater reduction compared to aerobic exercise. A review suggests that this might be a result of increased muscle mass and upregulation of glucose transporter type 4 (GLUT-4) (Saeidi et al., 2021). Both aerobic and resistance exercises can promote skeletal muscle contraction, leading to an increase in cytoplasmic calcium ions, which, through the activation of AMP-activated protein kinase (AMPK), translocate GLUT-4 to the cell membrane, thereby enhancing glucose uptake (Richter, 2021). In addition, resistance training has been shown to promote glycogen storage, while aerobic exercise enhances mitochondrial function and improves glucose utilization. These effects together help improve blood glucose control by reducing insulin resistance and enhancing glucose metabolism. Multi-component exercise may produce better outcomes in HbA1c intervention compared to single exercise modalities.

Given the substantial differences in intervention duration and frequency among the 19 included studies, this study conducted subgroup analyses and found that interventions lasting more than 6 months and involving exercise frequencies exceeding three times per week yielded more significant improvements in HbA1c levels. Sigal et al.’s (2007) study also indicated that multi-component exercise had twice the impact on HbA1c compared to aerobic exercise. This may be attributed to the reduction in overall sedentary time in older adults due to frequent exercise, which continuously induces exercise-related factors. Long-term adherence to exercise promotes muscular and skeletal health, enhances cardiorespiratory fitness, and improves metabolic efficiency. Additionally, the volume difference may be more effective than solely activating additional muscle groups (Houmard et al., 2004; Ishiguro et al., 2016). Over time, these sustained physiological changes may gradually improve blood glucose control, with exercise duration serving as a key determinant in enhancing insulin sensitivity in patients with T2DM (Li et al., 2012). Furthermore, long-term exercise may exert a continuous positive impact on insulin sensitivity. Increased insulin sensitivity helps lower blood glucose levels, thus improving HbA1c levels.

T2DM often coexists with abnormalities in lipid metabolism, manifested by decreased HDL levels and increased LDL and TG levels (Gong et al., 2016). This meta-analysis demonstrates a significant impact of multi-component exercise on improving HDL, LDL, and TG levels in patients with T2DM. Due to the integration of various exercise modalities, multi-component exercise differs from single exercise modalities in improving lipid metabolism patients with T2DM. Sigal et al. (2007), through a randomized controlled trial, confirmed that multi-component exercise intervention increased HDL by 0.04 mmol/L, decreased LDL by 0.11 mmol/L, and decreased TG by 0.26 mmol/L, whereas aerobic exercise intervention increased HDL by 0.01 mmol/L, decreased LDL by 0.16 mmol/L, and decreased TG by 0.09 mmol/L. Aerobic exercise primarily improves lipid composition by enhancing the activity of lipid metabolism-related enzymes (Egli et al., 2013). Lipoprotein lipase promotes the hydrolysis of TG, and prolonged aerobic exercise accelerates the oxidative breakdown of fats for energy substrates (Earnest et al., 2013). In multi-component exercise, not only does aerobic exercise play a role in T2DM intervention, but it also activates resistance intervention mechanisms. Resistance exercise regulates lipid metabolism processes by increasing the activity of lipid transport proteins (Augusto Libardi et al., 2012). Multi-component exercise may enhance the intervention effects on T2DM lipid metabolism by the interaction of various intervention mechanisms of different exercise modalities.

Given the significant heterogeneity in the intervention effects of multi-component exercise on T2DM lipid metabolism, subgroup analysis of the exercise duration in the included studies revealed that exercise duration was one of the sources of heterogeneity in multi-component exercise intervention in lipid metabolism. Subgroup analysis results suggest that an exercise duration of ≥6 months significantly improves lipid metabolism levels in patients with T2DM, possibly due to increased adaptability of T2DM to intervention mechanisms based on the interaction of multiple intervention mechanisms in multi-component exercise in improving lipid metabolism.

### Analysis of the overall effect of multi-component exercise on physical fitness in patients with T2DM

Research has found that the prevalence of sarcopenia in patients with T2DM is as high as 15.7%, and the risk of sarcopenia in individuals with T2DM is 2–4 times higher than in normal elderly individuals (Sarodnik et al., 2018). The coexistence of T2DM and sarcopenia can lead to adverse outcomes such as decreased muscle strength and functional impairment in patients (Anagnostis et al., 2020). Regarding the improvement of physical fitness in T2DM, this meta-analysis demonstrates that multi-component exercise promotes an increase in upper and lower limb muscle strength and peak oxygen consumption to improve physical function in patients with T2DM. The pooled results are statistically significant.

Multi-component exercise optimizes mitochondrial function in patients with T2DM, increases ATP production, and enhances oxygen utilization efficiency, thereby improving energy metabolism and meeting the high energy demands of muscle cells during exercise (Szendroedi, Phielix & Roden, 2011). Consequently, multi-component exercise increases peak oxygen consumption in patients with T2DM, improving cardiorespiratory function, which shares similar mechanisms with aerobic exercise in improving cardiorespiratory function in T2DM. However, in terms of intervention effects, Church et al.’s (2010) randomized controlled trial found that multi-component exercise intervention significantly increased peak oxygen consumption in patients, with an average increase of 1 ml/kg/min, while traditional aerobic exercise increased it by 0.5 ml/kg/min. This data suggests that multi-component exercise may be more effective than single exercise modalities in improving cardiorespiratory function. This study confirms the significant impact of multi-component exercise on improving upper and lower limb muscle strength in patients with T2DM. An experiment by Lambers et al. (2008) found that compared to single aerobic exercise, multi-component exercise not only did not improve upper limb strength but also decreased it by 0.3 kg. Multi-component exercise improved lower limb strength by 15.1 kg compared to aerobic exercise alone, while aerobic exercise alone increased lower limb strength by only 0.8 kg. This may be attributed to the activation of the PI3K/Akt pathway by resistance exercise combined with aerobic exercise (Wu & Ballantyne, 2017), increasing muscle oxygen consumption, increasing muscle capillaries, inducing muscle protein synthesis, optimizing skeletal muscle mass and function. Additionally, microRNAs play a crucial role in regulating mitochondrial function and muscle health, and aerobic exercise can improve mitochondrial dysfunction by regulating microRNA expression, thereby affecting muscle strength and endurance in T2DM (Long et al., 2023). Compared to single exercise modalities (aerobic exercise, resistance exercise), multi-component exercise provides an efficient strategy for improving physical fitness in patients with T2DM by integrating the intervention effects of different exercise modalities on muscle strength.

### Analysis of the overall effect of multi-component exercise on cognitive function in patients with T2DM

Long-term hyperglycemia can impair both brain function and peripheral nerves, thus affecting cognitive function (Alosco et al., 2012). Studies have confirmed (Kotsani et al., 2018) that patients with T2DM are at a higher risk of cognitive impairment compared to healthy individuals, and this risk is more significant in elderly patients with T2DM. Therefore, effective measures should be taken to delay and prevent the decline in cognitive function associated with T2DM. This meta-analysis indicates that multi-component exercise significantly improves overall cognitive function in patients with T2DM, with pooled results being statistically significant. A review suggests that growth factors, neuroplasticity, vascular function, among others, form the basis for exercise-induced improvements in cognitive function (Quigley, Mackay-Lyons & Eskes, 2020). Compared to single exercise modalities, multi-component exercise may improve overall cognitive function in T2DM by optimizing multiple brain mechanisms. Martínez-Velilla et al. (2021) demonstrated a significant improvement in overall cognitive function in T2D through a multi-component exercise regimen combining aerobic, resistance, and balance exercises. Aerobic exercise (Lan et al., 2018) can increase the levels of β-HB, inducing the transcription of neurotrophic factors, further promoting hippocampal neuroplasticity. Additionally, aerobic exercise can promote the release of anti-inflammatory substances from muscles (Hopps, Canino & Caimi, 2011), reducing the function of pro-inflammatory factors and thus improving cognitive function in T2DM. In contrast, resistance exercise tends to increase IGF-1 levels (Tsai et al., 2018), further promoting metabolic and functional improvements in the body. Balance ability has been shown to have a positive relationship with cognitive function (Meunier et al., 2021), and balance training can affect cognitive function improvement by improving balance in patients with T2DM. In summary, multi-component exercise, by integrating various intervention mechanisms from different exercise modalities, may maximize improvements in cognitive function in patients with T2DM.

### Analysis of the overall effect of multi-component exercise on quality of life in patients with T2DM

This study further explored the impact of multi-component exercise on the quality of life in patients with T2DM. The results indicate that multi-component exercise significantly improves vitality, mental health, physical functioning, and overall health perception in patients with T2DM. The improvement in quality of life is mediated by changes in body composition and is associated with improvements in blood glucose levels (Cai et al., 2017). Regular exercise enhances insulin sensitivity and reduces inflammation, contributing to better physical health and a reduction in diabetes-related complications (Zaki, Sharma & Vats, 2024; Annibalini et al., 2017). Mechanistically, aerobic exercise increases endorphin levels and reduces stress hormones, thereby improving mood and mental health (Ren & Xiao, 2023). On the other hand, resistance training enhances muscle strength and endurance, improving physical functioning and reducing fatigue (Ren & Xiao, 2023; Gacesa, Klasnja & Grujic, 2013). These physiological changes collectively contribute to the improvement of quality of life. Additionally, multi-component exercise, by combining various forms of exercise (aerobic, resistance), involves more tissues, organs, and systems, potentially leading to greater improvements in T2DM patients’ quality of life. However, this review also indicates that multi-component exercise did not have a significant impact on emotional role functioning, social functioning, physical role functioning, or pain perception. This may be attributed to factors such as the exercise environment, duration, types of multi-component exercise, and the quality and quantity of the relevant studies. Future research can further explore these aspects to better understand the comprehensive effects of multi-component exercise on the quality of life in patients with T2DM.

Moreover, some studies indicate that multi-component exercise not only outperforms single-mode exercise in improving HbA1c levels and inflammatory markers in patients with T2DM (Schwingshackl et al., 2014), but its diversified formats also enhance the intervention’s appeal and patient engagement (Batrakoulis et al., 2021). This is crucial for ensuring long-term exercise adherence. Additionally, the core components of multi-component exercise (such as walking, bodyweight resistance training, balance exercises, and flexibility activities) generally have low requirements for space and equipment, making them highly accessible and feasible. This allows for flexible implementation and promotion of such interventions across various settings, including community health programs, home self-management plans, and clinical pathways in hospitals or rehabilitation centers. The combination of higher adherence and implementation feasibility ensures that multi-component exercise can continue to deliver its positive health benefits—such as improvements in metabolism, physical fitness, and cognitive function—in real-world environments.

However, despite the benefits of multi-component exercise in enhancing adherence, several challenges remain, such as time constraints, physical discomfort caused by T2DM-related complications, and lack of motivation. To ensure the practical feasibility and sustained benefits of multi-component exercise, future strategies should focus on simplifying home-based exercise routines, developing personalized programs with gradual progression, providing professional guidance, utilizing group-based social support, and leveraging technological tools. Overcoming these barriers is crucial for translating strong evidence into meaningful improvements in the daily lives of patients with T2DM.

This study was conducted in strict accordance with the PRISMA statement checklist, yet it still has some limitations. (1) The literature search did not include unpublished documents, and some articles could not be included due to incomplete outcome data, which may affect the comprehensiveness of the information; (2) Some data in the study were merged using SMD due to inconsistencies in units or measuring instruments, which may affect the precision of the results; (3) Some studies did not implement blinding or did not specify whether blinding allocation methods were used, which could increase the risk of bias; (4) Due to significant variations in the reporting of exercise intensity across the included studies, subgroup analysis could not be performed. This limitation may affect the generalizability of the results and obscure the potential impact of exercise intensity on the effectiveness of multi-component exercise interventions.

## Implications for future research

To clarify the differential benefits of different exercise intensities for patients with T2DM, future studies should conduct standardized RCTs to investigate the ideal exercise intensity and combination of multi-component exercises, while standardizing the reporting of exercise intensity using consistent metrics. Additionally, studies should extend the follow-up period to assess the long-term effects of multi-component exercise and track exercise adherence. Furthermore, subgroup analyses should be conducted for patients with T2DM who have different disease durations, ages, and complications, and personalized exercise prescriptions should be developed.

## Conclusion

This meta-analysis demonstrates that multi-component exercise intervention effectively improves glycemic and lipid metabolism, physical fitness, cognitive function and quality of life in patients with T2DM, indicating its positive impact and practical value for patients with T2DM. Due to the unclear description of exercise intensity in the included literature, multi-component exercise mainly consisted of aerobic combined with resistance exercises. Therefore, this study did not conduct subgroup analysis on exercise intensity and mode in the included literature.

## Supplemental Information

10.7717/peerj.20146/supp-1 Supplemental Information 1 Contribution to Existing Knowledge.

10.7717/peerj.20146/supp-2 Supplemental Information 2 PRISMA 2010 checklist.

## Additional Information and Declarations

### Data Availability

The following information was supplied regarding data availability:

Raw data was not generated in this is a systematic review and meta-analysis.
