# Supplementary material for: Effectiveness of multi-component exercise in individuals with type 2 diabetes: a systematic review and meta-analysis
Source: PeerJ. 2025 Nov 20;13:e20146. doi: 10.7717/peerj.20146 (PMC12640639; doi:10.7717/peerj.20146)
Supplement: Supplemental Information 1 [file peerj-13-20146-s001.docx]

**Effectiveness of multi-component exercise in people with diabetes: A systematic review and meta-analysis**

**1. Limitations of Existing Research and the Positioning of This Study**

Previous meta-analyses on the effects of multi-component exercise in type 2 diabetes (T2DM) have the following gaps:

**1.1 Simplified Control Design:** Most studies focused on comparisons between MCE and single-modality exercise (aerobic or resistance alone), lacking high-quality evidence synthesis of MCE versus blank control (no intervention/low-intensity activities).

**1.2 Fragmented Outcome Dimensions:** Existing reviews were often limited to single domains such as glucose-lipid metabolism or physical fitness, failing to systematically evaluate the comprehensive benefits of MCE on metabolic, physical, and cognitive outcomes.

**1.3 Unclear Moderating Effects of Exercise Parameters:** The impacts of exercise frequency (≥3 sessions/week) and duration (≥6 months) on outcomes were not adequately quantified, limiting clinical applicability.

**1.4 Methodological Rigor Deficiencies:** Some studies did not adhere to PRISMA guidelines or neglected publication bias testing, reducing the credibility of conclusions.

**2. Innovative Breakthroughs of This Study**

**2.1 The First Multidimensional Meta-Analysis Comparing multi-component exercise vs. Blank Control Cross-Domain Outcome Integration:**

This study is the first to simultaneously quantify the effects of multi-component exercise on glucose metabolism (HbA1c, fasting blood glucose), lipid metabolism (HDL, LDL, TG), physical fitness (muscle strength, peak oxygen uptake, BMI), and cognitive function in T2DM patients, filling the gap in combined "metabolic-physical-cognitive" analyses.

Precision Comparison with Blank Control: By strictly including RCTs with blank control groups only (stretching/relaxation or routine care groups), this study eliminates interference from single-modality exercise and clarifies the independent net effect of MCE (HbA1c reduction: Standardized Mean Difference [SMD] = −0.52, P < 0.001).

**2.2 Refined Analysis of Exercise Parameters**

**HbA1c Improvement:** Optimal effects were achieved with interventions lasting ≥6 months and performed ≥3 times/week (SMD = −0.59 vs. −0.45 for <6 months, P < 0.001).

**Lipid Metabolism Modulation:** Long-term interventions (≥6 months) significantly enhanced HDL elevation (SMD = 0.34 vs. 0.27 for short-term) and LDL/TG reduction (SMD = −0.27/−0.21).

**Clinical Prescription Guidance:** Provides strong evidence supporting the multi-component exercise regimen of "≥3 sessions/week for ≥6 months."

**2.3 Demonstration of Methodological Rigor**

**Dual Compliance with PRISMA and Cochrane:** Strict adherence to PRISMA reporting standards and Cochrane risk-of-bias assessment tools (31 low-bias-risk studies) enhanced result credibility.

**Robustness Verification:** Sensitivity analyses (fixed/random-effects model consistency) and Egger’s test (P = 0.137) excluded publication bias, ensuring robust conclusions.

**3. Addressing and Correcting Field Controversies**

**Controversy Over "No Cognitive Benefits of multi-component exercise":** Previous small-sample studies reported inconsistent conclusions. This study, with a large sample (n = 586), confirms that multi-component exercise significantly improves overall cognition (SMD = 0.34), suggesting synergistic neuroregulatory effects from balance/resistance training.

**Misconception of "Irrelevant Exercise Duration":** Refutes the view that short-term interventions (≤3 months) suffice for lipid metabolism improvement, proving that ≥6 months is necessary for optimizing HDL/LDL/TG.

**4. Clinical and Research Implications**

**Clinical Practice:** Recommends multi-component exercise as a standard non-pharmacological intervention for T2DM, particularly for patients with metabolic abnormalities, sarcopenia, or high cognitive decline risk.

**Research Optimization:** Future trials should standardize reporting of exercise intensity and specific modality combinations (resistance-aerobic-balance ratios) to support in-depth subgroup exploration.

**5. Conclusion**

This study, through multidimensional outcome integration and rigorous methodological design, establishes the comprehensive benefits of multi-component exercise versus blank control in T2DM management. The findings provide critical evidence for updating international guidelines (ADA/WHO exercise recommendations) and advance the paradigm of "precision exercise prescription."
